# Non‐Respiratory Extracellular Electron Transfer Competes with Nitrogenase for Electrons in *Rhodopseudomonas Palustris*

**DOI:** 10.1002/advs.202501376

**Published:** 2025-05-14

**Authors:** Xuewen Liu, Panqing Qi, Weipeng Fan, Wuyang Liu, Xingjiang Li, Yong Nie, Xiao‐Lei Wu

**Affiliations:** ^1^ College of Engineering Peking University Beijing 100871 China; ^2^ Institute of Ecology Peking University Beijing 100871 China; ^3^ College of Life Sciences Qufu Normal University Qufu 273165 China; ^4^ School of Food and Biological Engineering Hefei University of Technology Hefei 230009 China

**Keywords:** anode reduction, biological nitrogen fixation (BNF), extracellular electron transfer (EET), photosynthetic bioelectrochemical nitrogen fixation system, Rhodopseudomonas palustris TIE‐1

## Abstract

Biological nitrogen fixation (BNF) is a pivotal process that reduces nitrogen to ammonium within the nitrogen cycle. Extracellular electron transfer (EET) between diazotrophs and the extracellular environment influences the occurrence and efficiency of BNF. Although extracellular electron acceptors can function as a component of the electron transport chain, providing energy for chemotrophic nitrogen fixation via extracellular respiration, the function and mechanism of outward EET in photosynthetic diazotrophs remain unclear. Here, using *Rhodopseudomonas palustris* TIE‐1, a photosynthetic bioelectrochemical nitrogen fixation system is established for simultaneous nitrogen fixation and current generation, to dissect the complex interaction between these two processes. Outward EET functions are found to maintain redox balance, rather than serving as an extracellular respiration pathway. It significantly suppresses BNF by competing with nitrogenase for electrons. Lumichrome serves as the primary electron shuttle for indirect electron transfer, while cytochromes play an important role in direct electron transfer. Notably, the *pio* operon participates in outward EET. This study reveals the interaction mechanism between photosynthetic BNF and outward EET, providing new insight into the regulatory mechanisms of nitrogen fixation in anoxygenic phototrophs across diverse environmental conditions.

## Graphical abstract

This study develops a photosynthetic bioelectrochemical nitrogen fixation system for simultaneous nitrogen fixation and current generation using *Rhodopseudomonas palustris*. Notably, extracellular electron transfer (EET) serves as a redox balancing strategy, suppressing nitrogen fixation by competing with nitrogenase for electrons. Lumichrome and cytochromes facilitate indirect and direct EET pathways, respectively.

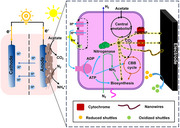

## 1 Introduction

Biological nitrogen fixation (BNF) is a microbial process in which atmospheric N_2_ is converted into bioavailable nitrogen by nitrogenase in specific bacteria and archaea.^[^ ^1^ ^]^ This process serves as the primary natural pathway for introducing N_2_ into biogeochemical cycles, accounting for nearly two‐thirds of global nitrogen fixation.^[^ ^2^ ^]^ Diazotrophs conventionally utilize chemical or light energy to perform BNF while reducing intracellular electron sinks such as oxygen or nitrate.^[^ ^3^ ^]^ The widespread presence of redox‐active minerals (such as those containing iron or manganese) in natural environments has fostered the evolution of complex mineral‐microbe interactions in diazotrophs.^[^ ^4^ ^]^ Specifically, under certain external pressures (e.g., redox stress or limited soluble electron acceptors/donors), these minerals can act as sources or sinks for extracellular electrons, supporting microbial growth through extracellular electron transfer (EET) processes.^[^ ^4^, ^5^ ^]^ EET is a crucial environmental adaptation strategy for microbes, enabling the regulation of energy metabolism, gene expression, and cellular physiology through electron exchange between living cells and the extracellular environment.^[^ ^5^, ^6^, ^7^, ^8^ ^]^

Bioelectrochemical systems (BESs) leverage controlled electrode potentials to simulate the redox properties of minerals, providing a simplified platform for studying EET between microbes and minerals.^[^ ^9^ ^]^ Bioelectrochemical nitrogen fixation (e‐BNF) employs electroactive diazotrophs as biocatalysts to drive nitrogen fixation through EET pathways, which represents not only a green nitrogen fixation technology, but also a simplified model for investigating EET mechanisms in diazotrophs.^[^ ^10^ ^]^ While extracellular electron donor‐driven BNF processes have been extensively characterized,^[^ ^10^, ^11^, ^12^, ^13^ ^]^ recent advances reveal that chemotrophic diazotrophs (such as *Geobacter* and *Clostridium*) can utilize anodes as extracellular electron acceptors when they are deprived of intracellular electron acceptors, achieving simultaneous N_2_ fixation and current generation.^[^ ^14^, ^15^, ^16^ ^]^ In this typical interaction with anodes, electrodes integrate into microbial respiratory chains as terminal electron acceptors,^[^ ^17^, ^18^ ^]^ supporting oxidative phosphorylation and ATP synthesis for BNF. Therefore, outward EET emerges as an essential energetic driver of chemotrophic nitrogen fixation.^[^ ^14^ ^]^ Photosynthetic diazotrophs contribute to another vital part of BNF.^[^ ^19^ ^]^ Before the evolution of oxygenic photosynthesis, anoxygenic phototrophs were considered as the primary nitrogen fixers on early Earth.^[^ ^20^, ^21^ ^]^ Distinct from oxidative phosphorylation and oxygenic photosynthetic phosphorylation, which necessitate the transfer of electrons to designated electron sinks like oxygen, nitrate, or extracellular solid materials, a notable trait of these organisms is the decoupling of energy production from substrate electron transfer under illuminated anaerobic conditions. Specifically, light‐driven electrons generate ATP through a closed‐loop cyclic photosynthetic electron transport chain, while electrons from the oxidation of substrates provide the reducing power required for BNF.^[^ ^22^ ^]^ Thus, outward EET may fulfill distinct roles in anoxygenic photosynthetic nitrogen fixation process compared to chemotrophic or oxygenic photosynthetic diazotrophs. Nevertheless, the underlying processes and mechanisms remain largely unknown.

*Rhodopseudomonas palustris* is a purple non‐sulfur bacteria (PNSB) that are highly promising for development as a biocatalyst due to its versatile metabolism.^[^ ^23^ ^]^ It has been isolated from a wide variety of environments, including anaerobic aquatic environments, soils, sediments, rice straw, and leaf litter.^[^ ^24^ ^]^ Notably, *R. palustris* encodes three nitrogenase isozymes: molybdenum nitrogenase, vanadium nitrogenase, and iron‐only nitrogenase.^[^ ^25^ ^]^ It efficiently harnesses solar energy through cyclic photophosphorylation to generate substantial amounts of ATP necessary for BNF. Studies utilizing *R. palustris* in photosynthetic bioelectrochemical systems (photo‐BESs) have successfully achieved the conversion of solar energy into chemical or electrical energy. E.g., *R. palustris* strain DX‐1 has been reported to possess the ability to generate electricity in microbial fuel cells (MFCs),^[^ ^26^ ^]^ while *R. palustris* Azul can either receive electrons from a cathode or transport them to an anode.^[^ ^27^ ^]^ Additionally, strain CGMCC 1.2180 has been shown to perform interspecific electron transfer with *Methanosarcina barkeri* for methane production.^[^ ^28^ ^]^ These investigations demonstrate the ubiquity of outward EET capabilities among *R. palustris* strains.^[^ ^23^ ^]^

In this study, we employed *R. palustris* TIE‐1 to set up a photosynthetic bioelectrochemical nitrogen fixation (photo‐e‐BNF) system for simultaneous BNF and anode reduction, with the aim to explore the interaction between photosynthetic N_2_ fixation and outward EET, along with elucidating the fundamental mechanisms underlying these processes. We systematically investigated the performance of the photo‐e‐BNF systems and revealed the unusual function of EET in *R. palustris* TIE‐1. To determine the relationship between BNF and EET, we genetically altered the electron flux network and compared the EET response to the modified network. Furthermore, a combination of electron microscopy, bioelectrochemical, molecular biological, and transcriptomic approaches was used to investigate the mechanism of electron transfer to the anode. Overall, this study highlights the competitive interplay between BNF and outward EET under photosynthetic conditions, broadening our understanding of electron flow dynamics within anoxygenic phototrophs across diverse redox environments.

## 2 Results

### 2.1 Outward EET Inhibits Photosynthetic Nitrogen Fixation in *Rhodopseudomonas Palustris* TIE‐1

**Figure 1:**
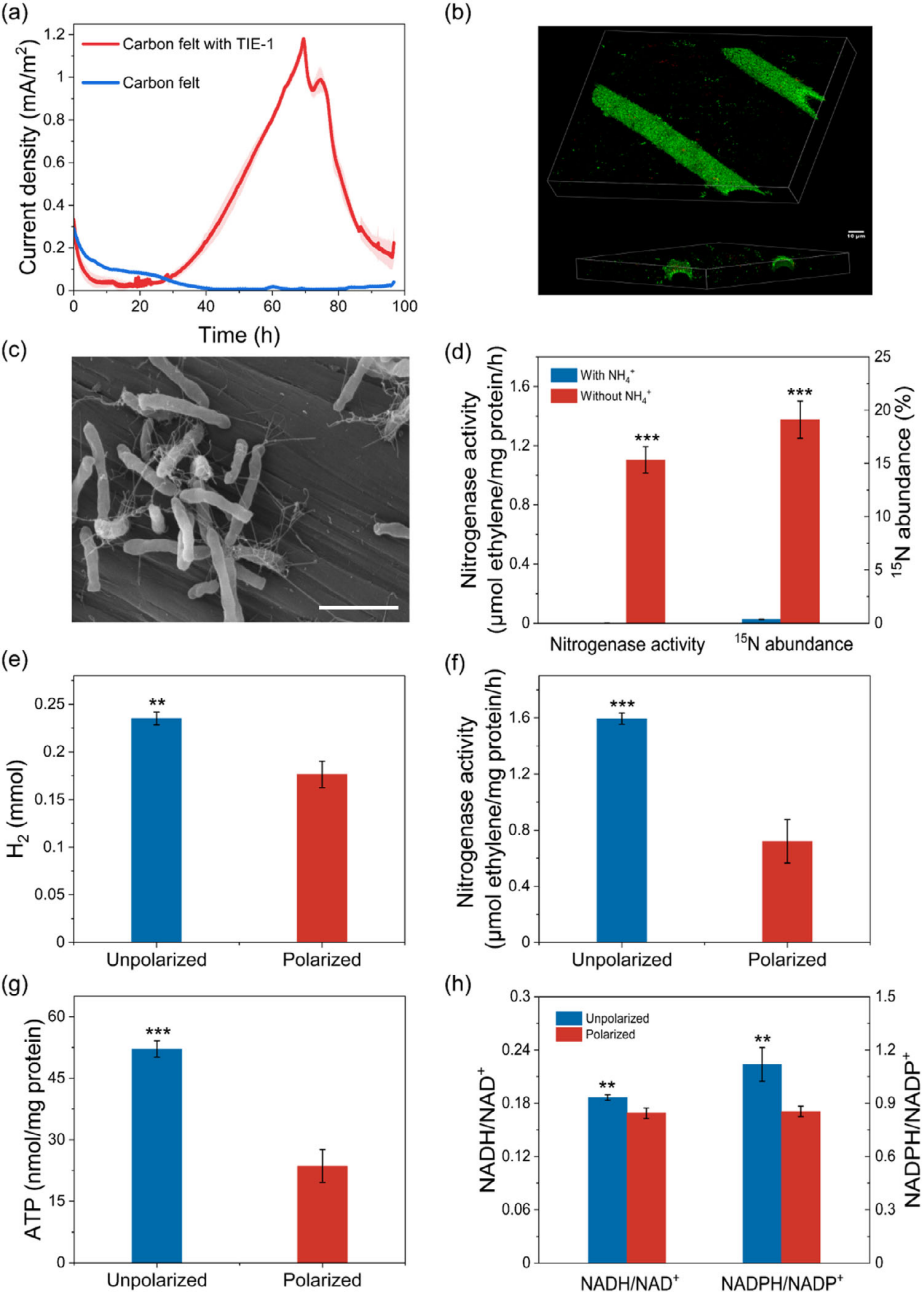
Current generation and BNF on the anode. a) Electricity generated by *R. palustris* TIE‐1 in photo‐e‐BNF systems. The shaded area represents the standard deviation. b) Biofilm of *R. palustris* on the anode. Cells were stained with LIVE/DEAD stain. c) SEM image of cells attached to the electrode surface. Scale bar, 3µm. d) ^15^N assimilation and nitrogenase activity of *R. palustris* in photo‐BESs with or without ammonium in the electrolyte. e) The production of H_2_, f) nitrogenase activity, g) intracellular ATP concentration, and h) the ratios of NADH/NAD^+^ and NADPH/NADP^+^ of WT*hupV^−^* cells incubated with or without poised anodes. Data were presented as mean ± SD (*n* = 3). Statistical analysis was performed using two‐tailed Student's *t*‐tests (d–h). ** *p *< 0.01, *** *p *< 0.001.

To determine whether *R. palustris* TIE‐1 could simultaneously fix nitrogen and generate current, a three‐electrode dual chamber reactor containing medium without fixed nitrogen was employed and incubated with TIE‐1 cells under illumination. With N_2_ as the sole nitrogen source and the anode poised at +0.3 V (vs. SCE), the photo‐e‐BNF systems exhibited distinct anodic current generation, reaching a maximum current density of ≈1.18 ± 0.01 mA m^−2^ (**Figure** **1**a). Over the course of three cycles, the systems maintained stable power generation while demonstrating shorter startup periods (Figure S1a, Supporting Information), consistent with the characteristic development of electroactive biofilms in BESs.^[^ ^29^ ^]^ Confocal laser scanning microscopy (CLSM) analyses suggested that cells attached to the anode surface with a thickness of ≈6 µm (Figure 1b). During SEM examination, many intricate extracellular filamentous structures were observed (Figure 1c), morphologically similar to those previously associated with dissimilatory Fe(III) oxide reduction in *R. palustris*.^[^ ^30^ ^]^ These observations confirmed EET from *R. palustris* TIE‐1 to the anode. The ^15^N labeling experiments revealed substantial nitrogen fixation activity, with the photo‐e‐BNF systems showing a marked ^15^N abundance of 19.10%, which was significantly higher than natural abundance levels (0.366%) (Figure 1d).^[^ ^31^ ^]^ Concurrent measurements of nitrogenase activity demonstrated a robust rate of 1.10 ± 0.09 µм ethylene (mg protein)^−1^ h^−1^ in the photo‐e‐BNF systems. These results provided evidence for current generation during N_2_ fixation on the anode by *R. palustris* TIE‐1.

To further explore the biochemical responses of BNF to anode reduction, we conducted comparative experiments in photo‐e‐BNF systems under both polarized and unpolarized conditions. Given that the H_2_ concomitantly produced during BNF could serve as an additional electron donor for the nitrogen fixation process (Figure S2, Supporting Information), we introduced a frameshift mutation in the *hupV* gene of *R. palustris* TIE‐1 to suppress the expression of the nickel‐iron uptake hydrogenase (WT*hupV* ^−^), as previously described.^[^ ^32^ ^]^ Notably, while WT*hupV* ^−^ showed similar biomass accumulation under both conditions (Figure S3, Supporting Information), polarized cultures demonstrated significantly reduced hydrogen production (0.18 ± 0.01 mм) compared to the unpolarized controls (0.24 ± 0.005 mм) (Figure 1e). This indicated diminished N_2_ fixation under polarized conditions, as nitrogenase stoichiometrically generates one H_2_ molecule for every N_2_ reduced to ammonia (Equation 1). Correspondingly, nitrogenase activity in polarized systems decreased to 0.72 ± 0.13 µм ethylene (mg protein)^−1^ h^−1^, representing a 1.21‐fold reduction versus controls (Figure 1f). BNF is an energy‐intensive process, requiring significant amounts of ATP and reducing power (Equation 1).^[^ ^33^ ^]^ As illustrated in Figure 1g, ATP generation was significantly reduced in polarized cells (23.58 ± 3.27 nм (mg protein)^−1^) compared to the unpolarized control (52.14 ± 1.64 nм (mg protein)^−1^). The NADH/NAD^+^ and NADPH/NADP^+^ ratios were also lower in polarized cells, with values of 0.17 ± 0.005 and 0.85 ± 0.02, respectively, compared to unpolarized control at 0.19 ± 0.002 and 1.12 ± 0.08, respectively (Figure 1h). These data suggested that polarized conditions were not favorable for BNF, as they prevented cells from accumulating sufficient ATP and reducing power, both of which are essential for nitrogen fixation. Collectively, these results indicated that outward EET had an inhibitory effect on BNF.

(1) $$\begin{array}{c} N_{2}+8H^{+}+16\mathrm{MgATP}+8e^{-}\to 2NH_{3}+H_{2}+16\mathrm{MgADP}+16\mathrm{Pi} \end{array}$$

### 2.2 Outward EET Functions as a Redox Balancing Strategy of *R. Palustris*

**Figure 2:**
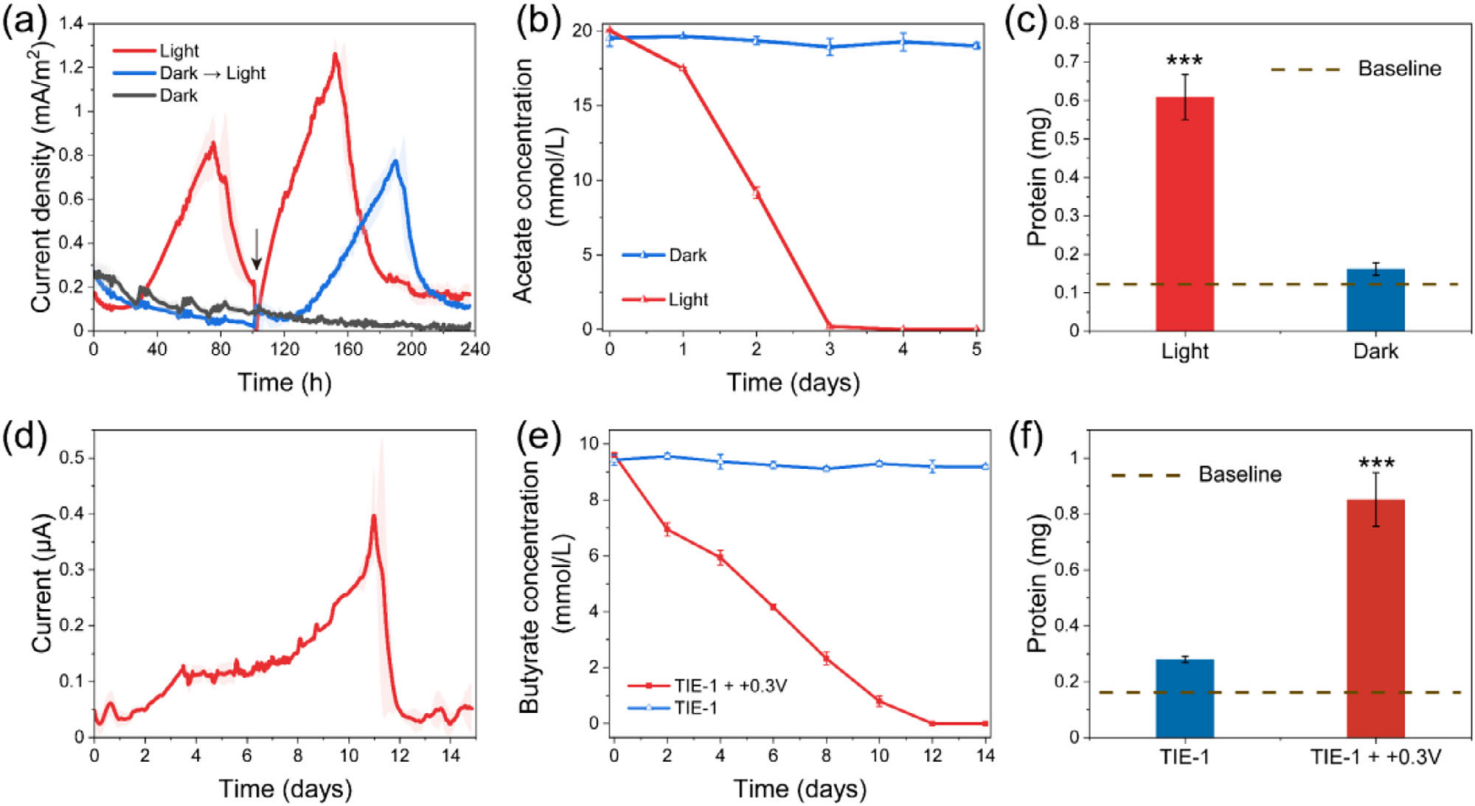
Electrochemical and metabolic performance of *R. palustris* TIE‐1 incubated at the anode under varying illumination regimes and substrate availability conditions. a) Current curves of BESs under illuminated, dark, and reintroduction of light conditions. The black arrow indicates the introduction of light for one of the dark groups and the replacement of fresh electrolytes for the illuminated group. b) Acetate consumption of BESs under illuminated and dark conditions. c) The total protein content of the BESs with or without illumination. The baseline represents the initial protein content. d) Current curves at +0.3 V (vs. SCE) in the electrolyte with butyrate under illuminated conditions. e) Butyrate consumption of photo‐BESs under polarized or unpolarized conditions. f) The total protein content of the photo‐BESs in the presence of poised and unpoised anodes. The baseline represents the initial protein content. Data were presented as mean ± SD (*n* = 3). Statistical analysis was performed using two‐tailed Student's *t*‐tests (c,f). *** *p *< 0.001.

Although *R. palustris* TIE‐1 could reduce the anode in photo‐e‐BNF systems, the absence of a polarized anode did not affect the growth of cells as compared with typical electricigens (e.g., *Geobacter* and *Shewanella*) (Figure S4, Supporting Information), suggesting that microbial growth and anode reduction are independent processes. To explore whether the anode could act as an electron acceptor for respiration in *R. palustris*, e‐BNF systems were operated under both dark and illuminated conditions. Complete light exclusion was achieved by wrapping reactors in tin foil. Current generation exhibited an absolute light dependence, with detectable anodic current observed exclusively under illumination (**Figure** **2**a). This phototrophic requirement was further confirmed through a parallel experiment where e‐BNF systems maintained in complete darkness for 100 h only initiated current production upon light exposure (Figure 2a). Notably, the electrons required for outward EET in *R. palustris* were not derived from the photosynthetic electron transport chain, as evidenced by the absence of detectable current under illuminated conditions in the absence of exogenous electron donors (Figure S5, Supporting Information). These findings highlight the role of light as an energy source in driving current generation. Additionally, under dark conditions, there was no detectable acetate consumption and negligible cellular growth (Figure 2b,c; Figure S6, Supporting Information). These data clearly demonstrated that anode reduction by *R. palustris* TIE‐1 in photo‐e‐BNF systems did not support traditional anaerobic extracellular respiration, as that was unrelated to energy generation.

During the anaerobic photoheterotrophic growth of PNSB, reducing equivalents from the oxidation of organic carbon sources must be consumed to maintain redox balance.^[^ ^34^ ^]^ We postulated that the anode in photo‐e‐BNF systems functioned as an extracellular electron sink to accept the extra reducing equivalents that could not be put toward biosynthesis. To verify this hypothesis, we used butyrate as a carbon source in the anodic chamber and added (NH_4_)_2_SO_4_ to prevent H_2_ production. Previous research has reported that the growth of PNSB on butyrate, a compound more reduced than biomass, required either NaHCO_3_ or DMSO, unless H_2_ was produced.^[^ ^34^ ^]^ As expected, in photo‐BESs with the anode polarized at +0.3 V (vs. SCE), the system produced an anodic current with a peak of ≈0.4 µA (Figure 2d). Simultaneously, butyrate in the photo‐BESs was completely consumed by *R. palustris* TIE‐1 within 12 days (Figure 2e), indicating that strain TIE‐1 was able to utilize the anode as an electron acceptor for growth on butyrate. In contrast, substrate consumption and cell growth were not observed in the unpolarized groups (Figure 2e,f). These results suggested that reduction of the anode by *R. palustris* TIE‐1 served as a redox balancing strategy during anaerobic photoheterotrophic growth.

### 2.3 Outward EET Competes with Nitrogenase for Electrons

**Figure 3:**
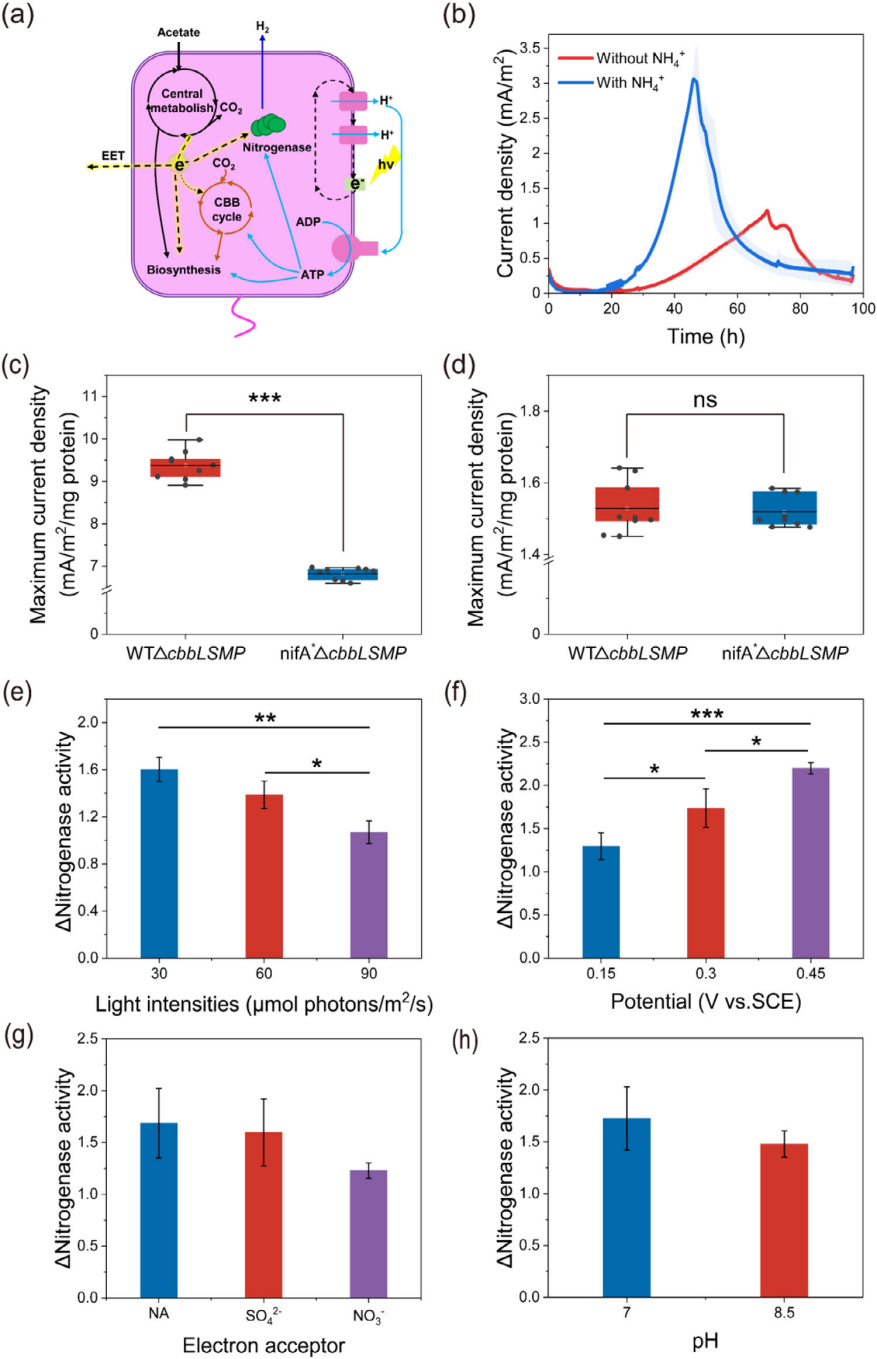
Analysis of the relationship between nitrogen fixation and anode reduction. a) Redox balancing strategies of *R. palustris* TIE‐1 in the photo‐e‐BNF systems. b) The average current densities of photo‐BESs with or without ammonium in the anolyte. The shaded area represents the standard deviation. The normalized maximum current density generated through c) direct and d) indirect pathways. e) The impact of light intensity, f) redox potential, g) the presence of other electron acceptors, and h) pH on the interaction between nitrogen fixation and anode reduction. Data were presented as mean ± SD (*n* = 3). Statistical analysis was performed using two‐tailed Student's *t*‐tests (c–h). * *p *< 0.05, ** *p *< 0.01, *** *p *< 0.001, ns, no significance.

Nitrogenase in PNSB is considered the primary electron sink under nitrogen‐fixation conditions, aside from biosynthesis.^[^ ^34^ ^]^ Based on our results, in photo‐e‐BNF systems, reduction of the anode via EET and the production of H_2_ by nitrogenase served as extracellular and intracellular redox‐balancing mechanisms, respectively (**Figure** **3**a). To further investigate whether anode reduction competed with BNF for electrons, we initially operated photo‐BESs both with and without ammonium. When grown in N‐sufficient electrolyte, *R. palustris* TIE‐1 generated a current density with a maximum of ≈3.06 ± 0.2 mA m^−2^ within 48 h. This performance was notably swifter and higher compared to the N‐deficient environment, in which the maximum current density reached only 1.19 ± 0.003 mA m^−2^ over 74 h (Figure 3b). Notably, N‐sufficient conditions supported substantially greater intracellular electron flux toward biosynthesis, as evidenced by greater biomass production in the absence of nitrogen fixation (Figure S7a, Supporting Information). Interestingly, despite this increased biosynthetic activity, the extracellular current generated under N‐sufficient conditions consistently exceeded that under N‐deficient conditions. This observation implied that a substantial portion of substrate electrons was utilized for BNF, consequently restricting electron availability for other cellular metabolic processes. To negate the impact of growth rate (Figure S7b, Supporting Information), (NH_4_)_2_SO_4_ was introduced into the photo‐e‐BNF systems after 48 h of operation to inhibit the activity of nitrogenase. The incorporation of ammonium resulted in a steeper slope of the current curve, ultimately causing an increase in the maximum current density (Figure S7c, Supporting Information). This suggests that a competition for electrons may exist between anode reduction and nitrogen fixation.

However, under N‐sufficient conditions, the Calvin‐Benson‐Bassham (CBB) cycle served as the primary electron sink in *R. palustris*. Despite a significant reduction in CBB cycle flux triggered by H_2_ production in nitrogen‐fixation cells, this process did not entirely shut down.^[^ ^34^ ^]^ To eliminate the impact of the CBB cycle and to better understand the competition for electrons between nitrogenase and extracellular anodes, a ribulose 1,5‐bisphosphate carboxylase‐ and phosphoribulokinase‐deficient mutant strain (WTΔ*cbbLSMP*) was constructed to block electrons from entering the CBB cycle.^[^ ^35^ ^]^ Meanwhile, a nifA^*^Δ*cbbLSMP* mutant was established to allow nitrogenase genes to express constitutively even in NH_4_^+^‐containing medium.^[^ ^34^ ^]^ This enabled us to evaluate the effect of the modified electron flux network on EET performance under controlled conditions. Given that most cells in the photo‐BESs exist in a planktonic state (Figure S8a, Supporting Information), we employed dialysis bag‐shielded electrodes in a parallel experimental setup to assess the potential contribution of planktonic cells to current generation. Quantitative analysis of NH_4_^+^‐amended photo‐BESs showed that both mutant strains exhibited comparable indirect current levels, contributing 49.56% ± 2.5% and 38.44% ± 1.76% of the total current for WTΔ*cbbLSMP* and nifA^*^Δ*cbbLSMP*, respectively (Figure S8b, Supporting Information). However, in unshielded electrode configurations, the maximum current density generated by WTΔ*cbbLSMP* was 25.43% ± 3.86% lower than that of nifA^*^Δ*cbbLSMP* (Figure S8c, Supporting Information). This discrepancy likely stemmed from the reduced electrode surface colonization observed in WTΔ*cbbLSMP* cells (Figure S8a, Supporting Information). Considering the differential contributions of electron transfer pathways to EET, we proposed that planktonic cells primarily engage in indirect electron transfer, whereas attached cells were capable of both direct and indirect electron transfer to anodes. Accordingly, we normalized the experimental results using Equations (2) and (3) to account for pathway‐specific contributions. As expected, both mutants showed similar indirect EET performance. However, WTΔ*cbbLSMP* demonstrated significantly enhanced direct EET, achieving maximum current densities of 9.38 ± 0.34 mA m^−2^ (mg protein)^−1^ versus 6.88 ± 0.14 mA m^−2^ (mg protein)^−1^ for nifA^*^Δ*cbbLSMP* (Figure 3c,d). This divergence may stem from the similar total biomass levels of the two mutants in the BESs (Figure S8a, Supporting Information), reflecting comparable electron flux toward biosynthesis. Under these conditions, the sustained biosynthesis of endogenous electron shuttles remained largely unaffected. Therefore, blocking electron flow to nitrogenase likely increased the availability of electrons for extracellular transfer, particularly enhancing direct EET to the anode.

(2) $$Nj_{d}=\frac{j-j_{i}}{\mathrm{Anode}\;\mathrm{surface}\;\mathrm{biomass}}$$

(3) $$Nj_{i}=\frac{j_{i}}{\mathrm{Total}\;\mathrm{biomass}}$$

where *Nj_d_*, normalized current density of the direct electron transfer pathway;

*Nj_i_*, normalized current density of the indirect electron transfer pathway;

*j*, current density under non‐isolated conditions;

*j_i_*, current density under isolated conditions.

To further investigate the potential influence of dynamic environmental conditions on electron allocation between BNF and EET, we employed WT*hupV* ^−^ to systematically evaluate these competitive interactions across gradients of light intensity, redox potential, pH, and alternative electron acceptors. The degree of electron competition was quantified using ΔNitrogenase activity, defined as the ratio of nitrogenase activity under unpolarized conditions to that under polarized conditions. Elevated ΔNitrogenase activity values reflect greater nitrogenase suppression, indicating increased electron diversion away from nitrogenase. The results showed that ΔNitrogenase activity exhibited an inverse correlation with light intensity but a positive correlation with redox potential (Figure 3e,f). This suggested that elevated light intensities promote intracellular electron flux toward nitrogenase activation,^[^ ^36^ ^]^ while increased redox potentials strengthened the oxidizing capacity of the extracellular electrode, favoring EET. Additionally, the presence of common electron acceptors (5 mm sulfate or nitrate) showed no measurable effect on electron competition (Figure 3g), consistent with their metabolic irrelevance to *R. palustris* during photoheterotrophic growth.^[^ ^37^ ^]^ While pH extremes (≤5.5) completely inhibited bacterial growth, moderate variations (pH 7–8.5) had a negligible impact on the electron competition dynamics between EET and BNF (Figure 3h).

### 2.4 Lumichrome Serves as Electron Shuttle for Indirect Electron Transfer

**Figure 4:**
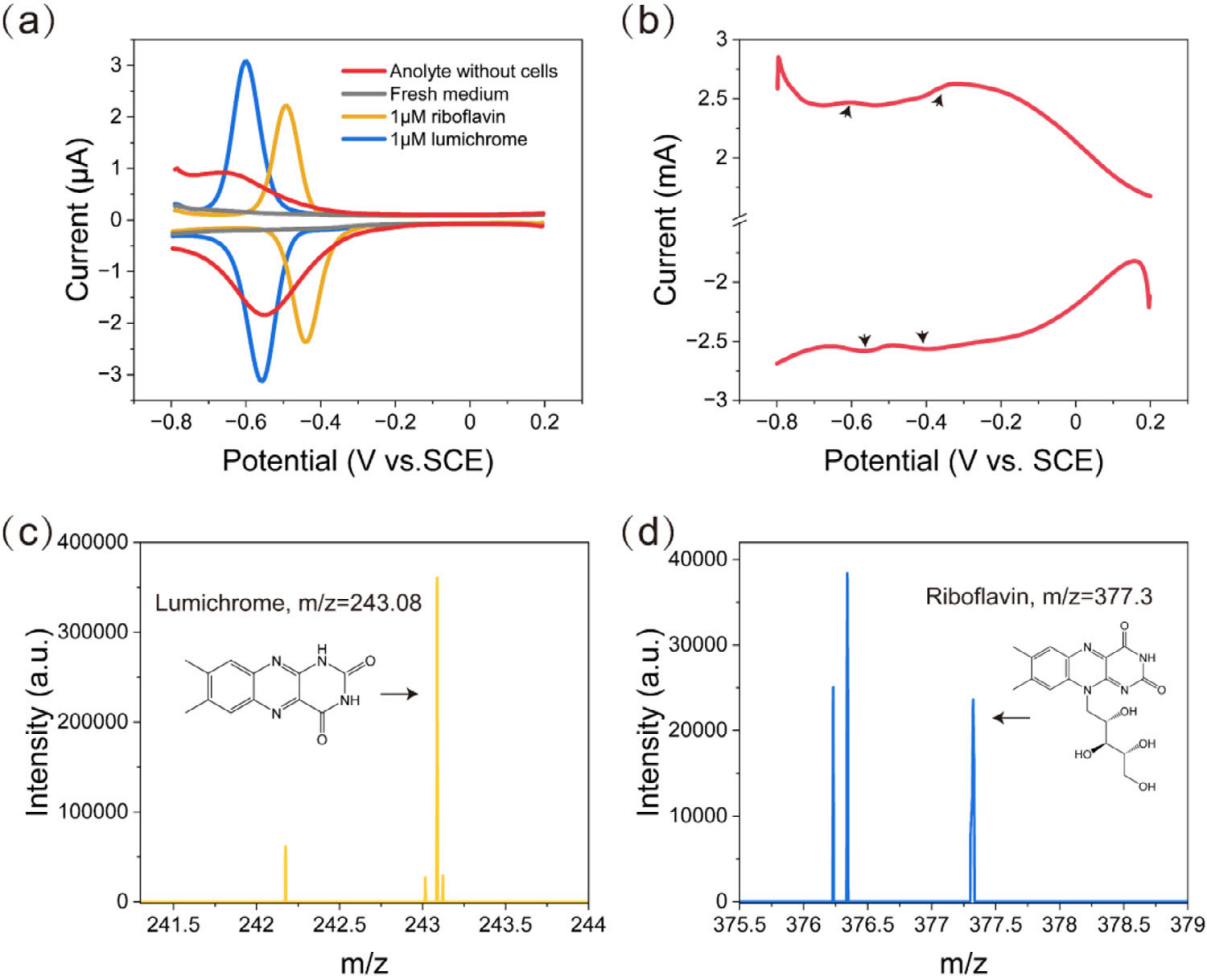
Direct and indirect electron transfer to the anode. a) DPV analyses of filtered (0.22 µm) cell‐free anolyte. b) DPV of the electrochemically incubated biofilm on the anode. Black arrows indicate the redox peaks. Mass spectrometry analysis of c) lumichrome and d) riboflavin in photo‐e‐BNF systems.

The detection of current in photo‐e‐BNF systems despite physical separation between cells and electrodes provided compelling evidence for the presence of electron shuttles (Figure S9a, Supporting Information). To identify potential redox mediators involved in *R. palustris* TIE‐1, DPV was conducted with filtered (0.22 µm) cell‐free media after current generation was achieved. Analysis of the DPV curves indicated reversible redox peaks at −0.6 and −0.55 V (vs. SCE), while no redox peaks were observed on the voltammograms obtained with fresh medium (**Figure** **4**a). This electrochemical feature closely resembled that of lumichrome, one of the photolysis products of riboflavin.^[^ ^38^, ^39^ ^]^ Comparative analysis revealed alignment between the redox peaks in the anolyte supernatant and those of lumichrome standard (Figure 4a). Subsequent LC‐MS analysis further confirmed lumichrome accumulation in the anolyte following current generation (Figure 4c; Figure S10a, Supporting Information). Notably, while riboflavin was detected in the anolyte, its mass spectrometry signal intensity was substantially weaker than that of lumichrome (Figure 4c; Figure S10a, Supporting Information). This result, combined with the absence of riboflavin redox signals in DPV measurements, suggested that most of the riboflavin secreted by *R. palustris* TIE‐1 had photolyzed to lumichrome.^[^ ^38^ ^]^ Thus, lumichrome emerged as the primary contributor to indirect electron transfer.

To better understand the role of lumichrome in EET, 1 µm lumichrome was added into the photo‐e‐BNF systems to monitor the current response.^[^ ^40^ ^]^ Chronoamperometry revealed that lumichrome addition significantly enhanced the maximum current density by 10.94% ± 5.91% (Figure S11a,b, Supporting Information). Moreover, this current stimulation occurred without concomitant biomass changes (Figure S11c, Supporting Information), consistent with previous findings in *R. palustris* GDM1.167.^[^ ^38^ ^]^ Therefore, the results suggested that lumichrome functions as an electron shuttle, facilitating EET from *R. palustris* to extracellular electron acceptors.

### 2.5 Cytochrome‐Mediated Direct Electron Transfer

DPV analysis of bioanodes revealed two distinct reversible redox couples (Figure 4b), which were absent in abiotic controls (Figure S9b, Supporting Information), suggesting the presence of redox‐active components on the electrode surface. Detailed electrochemical characterization showed that the redox pair located approximately at −0.6 V (vs. SCE) corresponded to lumichrome, while the other redox couples observed at −0.32 and −0.4 V (vs. SCE) were presumed to be redox‐active proteins of *R. palustris* TIE‐1. It has been extensively documented that outer‐membrane cytochromes (OMCs), particularly multiheme c‐type cytochromes, play a crucial role in mediating EET for the direct reduction of extracellular solids and electrodes in model EAMs, such as *S. oneidensis* and *G. sulfurreducens*.^[^ ^41^, ^42^ ^]^ Genomic analysis of strain TIE‐1 identified 24 cytochrome‐coding sequences containing characteristic heme‐binding motifs (CXXCH) (Table S3, Supporting Information). Notably, half of these protein sequences contained a signal peptide sequence, which is essential for translocation of the protein across the membrane and onto the cell surface. Among the putative multiheme cytochromes of strain TIE‐1 (*Rpal_0817*, *Rpal_1087*, and *Rpal_2214*, with a minimum of three heme‐binding motifs), *Rpal_0817* (*pioA*) has been previously characterized as essential for Fe(II) oxidation and cathodic electron uptake.^[^ ^43^, ^44^ ^]^

**Figure 5:**
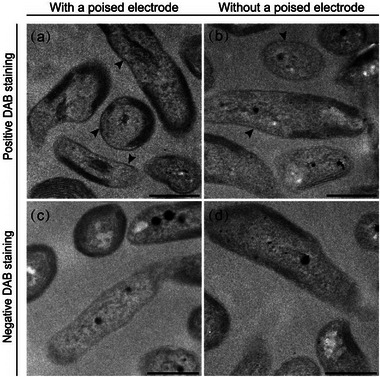
TEM visualization of DAB‐stained cells. a,c) Cells incubated with a poised electrode. b,d) Cells incubated with an unpoised electrode. a,b) Positive DAB staining with the addition of H_2_O_2_. c,d) Negative DAB staining without the addition of H_2_O_2_. The black arrows indicate the visible staining. Scale bar, 500 nm.

To assess the expression of cytochromes during anode reduction, the presence of heme‐binding cytochromes on the cell surface was examined through cytochrome reactive staining using 3,3'‐diaminobenzidine (DAB).^[^ ^45^ ^]^ TEM analysis of positive staining with DAB and H_2_O_2_ after incubation in photo‐e‐BNF systems revealed the presence of a dark layer along the outer edges of cells, while no visible staining was observed for cells treated with DAB alone (**Figure** **5**). This indicated the localization of heme‐binding proteins on the cell surface.^[^ ^46^ ^]^ Additionally, cells incubated under polarized conditions exhibited more intense staining on the cell surface compared to those incubated under unpolarized conditions, suggesting increased cytochrome expression under polarization.

**Figure 6:**
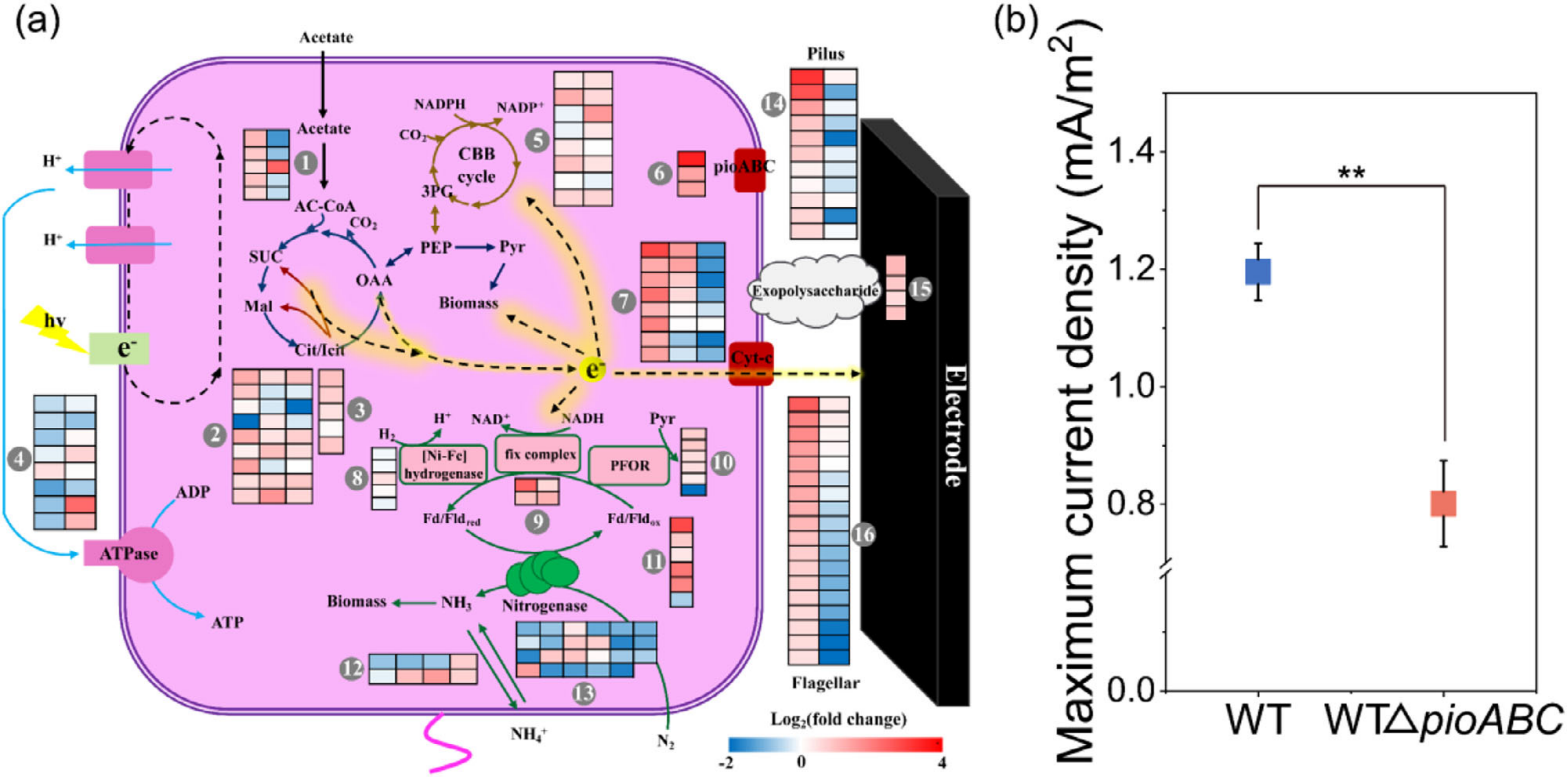
The mechanism of anode reduction during photosynthetic nitrogen fixation by *R. palustris* TIE‐1. a) Differential gene expression analysis of *R. palustris* TIE‐1 growing with a poised anode versus without a poised anode. Genes include those that encode (1) Acyl‐CoA synthetase, (2) enzymes participating in the tricarboxylic acid cycle, (3) enzymes participating in the Glyoxylate cycle, (4) ATP synthase, (5) enzymes participating in the CBB cycle, (6) *pio* operon, (7) c‐type cytochrome, (8) Ni/Fe‐hydrogenase, (9) fix complex, (10) PFOR, (11) ferredoxin and flavodoxin, (12) enzymes participating in the ammonium assimilation, (13) nitrogenase, (14) pilus, (15) exopolysaccharide, (16) flagellar. b) Maximum current density of WT and WTΔ*pioABC* mutant in photo‐e‐BNF systems. Data were presented as mean ± SD (*n* = 3). Statistical analysis was performed using two‐tailed Student's *t*‐tests (b). ** *p *< 0.01.

To gain further insight into gene expression during anode reduction in photo‐e‐BNF systems, the transcriptional responses of *R. palustris* TIE‐1 were compared for incubations under polarized and unpolarized conditions. In accordance with the competition for electrons between BNF and anode reduction, the genes responsible for BNF were found to be downregulated, while those associated with EET were upregulated. Here, genes involved in central carbon metabolism (glyoxylate and tricarboxylic acid cycles) exhibited consistent expression profiles regardless of polarization state, suggesting these metabolic pathways generated equiv. amounts of reducing equivalents under both experimental conditions (**Figure** **6**a; Tables S4 and S5, Supporting Information).^[^ ^34^ ^]^ Nitrogenase expression was significantly downregulated under polarization, aligning with the observed decreases in nitrogenase activity. Interestingly, electron‐transfer proteins, particularly the Fix complex and pyruvate‐flavodoxin oxidoreductase (PFOR),^[^ ^47^, ^48^ ^]^ were upregulated, possibly due to the presence of alternative electron acceptors. The downregulation of ATP synthase further indicated the suppression of BNF during anode reduction. Consistent with the observed localization of cytochromes (Figure 5), nearly half of all c‐type cytochrome genes were highly expressed during anode reduction (Figure 6a; Tables S4 and S5, Supporting Information), further supporting the involvement of c‐type cytochromes in direct electron transport processes. Additionally, the type IV pilus and flagella were more actively expressed under polarized conditions, consistent with the numerous nanofilaments observed on the anode surface (Figure 1c). Previous studies have indicated that the nanofilaments of flagella or pili in *R. palustris* possess conductive properties.^[^ ^30^ ^]^ Moreover, exopolysaccharide synthesis genes were highly upregulated in the anode biofilms. Exopolysaccharides are known to be an important component of extracellular polymeric substances (EPS), and the latter not only facilitate biofilm formation and adhesion, but may also serve as a transient medium for microbial EET.^[^ ^49^ ^]^ Therefore, both the nanofilaments and EPS of strain TIE‐1 are likely to participate in direct electron transfer to the anode.

Previous research has reported that the membrane protein complex PioAB and the periplasmic protein PioC play a direct role in the utilization of extracellular electrons for photoferrotrophic and photoautotrophic growth of *R. palustris*.^[^ ^43^, ^44^, ^50^ ^]^ Of note, the PioABC complex was significantly upregulated under polarized conditions (Figure 6a; Tables S4 and S5, Supporting Information), suggesting a potential involvement in outward EET. To investigate whether the PioABC system contributes to anode reduction, we constructed a *pioABC* knockout strain (WTΔ*pioABC*). As shown in Figure 6b, the maximum current density of WTΔ*pioABC* was 34.38% ± 6.71% lower than that of the WT. This observed difference was not attributed to alterations in biomass accumulation on the electrode surface or variations in electron shuttles within the systems (Figure S12, Supporting Information). Regardless, these results showed that the *pio* operon participates in electron output.

## 3 Discussions

Our study provides evidence for simultaneous nitrogen fixation and current generation by *R. palustris* TIE‐1 and reveals that anode reduction through direct and indirect EET suppresses photosynthetic nitrogen fixation.

The transfer of reducing equivalents, produced by microbial substrate metabolism, to extracellular acceptors in the form of electrons is common in nature. Current research predominantly views microbial EET as an extension of the intracellular respiratory chain into the external environment, where minerals or electrodes serve as terminal electron acceptors in ATP‐generating pathways.^[^ ^17^ ^]^ Consequently, the effective transfer of intracellular electrons to the extracellular electron acceptors is imperative for driving energy metabolism. However, since energy can originate from light, EET may play a distinct role in photosynthetic metabolism. Specifically, it has been reported that outward EET can assist oxygenic photosynthetic microalgae in alleviating photo‐oxidative stress under high light conditions.^[^ ^51^ ^]^ In this study, our results demonstrate that outward EET functions as a redox balancing strategy for anoxygenic phototrophs, rather than constituting an extracellular respiratory process (Figure 2). A previous study suggested that microbial EET is not limited to mineral‐respiring bacteria, but rather represents a fundamental aspect of microbial metabolism related to different environments.^[^ ^52^ ^]^ Therefore, this non‐respiratory mode of EET reflects an adaptive response to redox stress that enhances metabolic flexibility during complex substrate degradation. However, as EET did not have a decisive impact on the growth of *R. palustris*, anode reduction in photo‐e‐BNF systems was not mandatory. Most cells were present in the planktonic fraction, facilitating indirect electron transfer by secreting electron shuttles. This mechanism typically exhibits lower electron transfer efficiency compared to direct transfer pathways.^[^ ^53^ ^]^ Due to this inherent distinction, the photo‐e‐BNF systems based on *R. palustris* TIE‐1 exhibited a significantly lower current density compared to *G. sulfurreducens* fuel cells (0.17 ± 0.015 mA cm^−2^).^[^ ^14^ ^]^

The dynamic electron flow system regulates the transfer of electrons to various electron sinks, thereby driving energy metabolism.^[^ ^54^ ^]^ Our results revealed that outward EET inhibited BNF by competing with nitrogenase for electrons in *R. palustris* TIE‐1 cells. Transcriptome analysis demonstrated that the nitrogenase‐encoding genes were downregulated during anode reduction (Figure 6a; Tables S4 and S5, Supporting Information), aligning with previous observations of nitrogenase suppression in response to electron acceptors such as dimethyl sulfoxide or the expression of CBB cycle genes.^[^ ^55^, ^56^ ^]^ These results suggested that when alternative electron acceptors were available, the role of nitrogenase as an electron sink in redox balance would be restricted. Therefore, the interaction between photosynthetic nitrogen fixation and EET in *R. palustris* cells essentially involves the redistribution of substrate electrons, differing from the regulatory mechanisms governing substrate metabolism in *G. sulfurreducens*.^[^ ^14^ ^]^ Specifically, under N‐deficient conditions, substrate electrons were preferentially allocated to nitrogenase for BNF, while excess electrons were shifted away from nitrogen fixation toward extracellular transport. This pattern indicates that *R. palustris* likely prioritizes energy‐efficient redox balancing strategies, given that nitrogenase requires significant ATP support.

We also observed that the accumulation of CO_2_ in photo‐e‐BNF systems was significantly lower compared to conditions where nitrogen fixation occurred alone (Figure S13, Supporting Information). This meant an increased recycling of CO_2_ produced by the complete oxidation of substrates, as no CO_2_ or NaHCO_3_ was supplied to the cultures. Additionally, the Rubisco enzymes, which serve as the key enzymes of the CBB cycle, exhibited an up‐regulation trend throughout the photo‐e‐BNF process (Figure 6a; Tables S4 and S5, Supporting Information). It has been previously reported that the expression of Rubisco was influenced by the intracellular redox state, which was modulated by factors such as substrate redox reactions and the presence of external electron acceptors.^[^ ^57^, ^58^ ^]^ The activation of carbon fixation during the photo‐e‐BNF process may be explained by the inhibition of nitrogenase, which weakened its competition with the CBB cycle for electrons.^[^ ^34^ ^]^ These results suggest that there are mechanisms in place to regulate the allocation of electrons among different electron sinks within *R. palustris* cells.

Based on the understanding of intracellular electron flow dynamics in *R. palustris*, the performance and efficiency of the photo‐e‐BNF systems depend on the trade‐off between outward EET and BNF. Blocking electron flow to other metabolic processes that significantly consume reducing power (such as the CBB cycle or other biosynthetic pathways) can increase electron allocation toward EET and nitrogenase, thereby improving the overall performance of the photo‐e‐BNF systems. Strategies such as electrode surface modification, genetic engineering of electron transfer proteins, and synthetic biology approaches can enhance extracellular current production and harvesting.^[^ ^59^ ^]^ Furthermore, increasing light intensity (Figure 3e) or constructing microbe‐inorganic hybrid systems can enhance energy and electron supply to nitrogenase.^[^ ^36^, ^60^ ^]^ Appropriately reducing the potential (Figure 3f) or constructing a biocathode‐based photo‐e‐BNF system to induce nitrogenase to function as an intracellular electron sink could theoretically improve the overall nitrogen fixation efficiency, as research has demonstrated that strain TIE‐1 can utilize extracellular electrons from the cathode for metabolism.^[^ ^50^ ^]^

Previous studies on the EET of *R. palustris* TIE‐1 have primarily focused on biocathodes,^[^ ^44^, ^50^ ^]^ with insufficient attention given to its outward EET. We found that *R. palustris* TIE‐1 transported electrons to the anode through both direct and indirect pathways. Interestingly, the *pio* operon was involved in electron output, whereas previous studies had shown that it served as a crucial channel for electron uptake.^[^ ^44^ ^]^ This reversible EET capacity contrasts with previous observations made in the electric syntrophic coculture of *R. palustris* CGMCC 1.2180 and *M. barkeri*, wherein the *pio* operon was inactive in the interspecies electron transfer process.^[^ ^28^ ^]^ This discrepancy may be due to the different redox conditions experienced by the *R. palustris* cells, as the voltage applied to the photo‐e‐BNF system was higher. Additionally, our results indicated that *R. palustris* TIE‐1 indirectly reduced the anode by secreting riboflavin, with the photolysis product lumichrome, also serving as an electron shuttle, participating in indirect EET (Figure 4c,d), while the presence of the two electron shuttles was not detected during the inward EET.^[^ ^44^ ^]^ Prior research has established that riboflavin can exist in a free state or bind with extracellular cytochrome c to form flavocytochrome, thereby accomplishing electron transfer.^[^ ^61^, ^62^, ^63^ ^]^ For instance, in *S. loihica* PV‐4, the free riboflavin acted as an electron shuttle to stimulate outward EET, while bound riboflavin was involved in inward EET.^[^ ^64^ ^]^ The bidirectional electron transfer process mediated by riboflavin in *R. palustris* TIE‐1 may be similar.

Mineral‐microbe interactions are pivotal in the biogeochemical cycling of elements. Environmental minerals have diverse impacts on diazotrophs. Certain minerals serve as sources of bio‐essential elements or enzyme metal cofactors, or provide energy to diazotrophs via elemental redox reactions.^[^ ^65^, ^66^ ^]^ However, many metal‐containing minerals can increase intracellular ROS levels in diazotrophs through metal dissolution, release, and redox reactions, ultimately inhibiting the nitrogen fixation process.^[^ ^67^ ^]^ Our findings uncover a novel potential interaction mechanism wherein environmental oxidative minerals act as extracellular electron sinks, competing with nitrogenase for electrons and ultimately impeding anoxygenic photosynthetic nitrogen fixation. Given this, it seems that the efficiency of nitrogen fixation by anoxygenic phototrophs in oxidative environments has been potentially overestimated.^[^ ^68^ ^]^ Notably, these mineral‐captured electrons could contribute to a communal environmental electron pool. The resulting reduced minerals may then function as electron donors, shuttles, or electron‐storing “batteries”, supporting the growth of diverse autotrophs (such as metal‐oxidizing bacteria).^[^ ^5^, ^69^, ^70^ ^]^ This electron‐sharing network may drive long‐term ecological selection, shaping microbial community structure and function across evolutionary timescales.

## 4 Conclusion

In summary, our study provides a greater understanding of the outward EET pathways in *R. palustris* and their interaction mechanism with anoxygenic photosynthetic nitrogen fixation processes. Results suggest that the outward EET pathway in *R. palustris* functions primarily as an alternative redox‐balance strategy, rather than as an extracellular respiration process. Due to its functional redundancy with nitrogenase in maintaining redox balance, the outward EET pathway competes with nitrogenase for electrons, thereby inhibiting nitrogen fixation. Additionally, electrons are transferred extracellularly via lumichrome, which acts as an electron shuttle, and c‐type cytochromes. These results will facilitate a better understanding of the adaptation strategies of anoxygenic phototrophs in complex environments and provide a valuable foundation for future research on the EET mechanism and the rational engineering of *R. palustris*.

## 5 Experimental Section

### Bacterial Strains and Growth Conditions

All *R. palustris* strains were grown anaerobically in photosynthetic medium (PM) under an argon (Ar) headspace.^[^ ^71^ ^]^ For nitrogen‐fixing conditions, (NH_4_)_2_SO_4_ was omitted from the PM, and cells were grown under a N_2_ headspace (NFM). Acetate or butyrate was used as a carbon source at final concentrations of 20 mm or 10 mm, respectively. For the studies described here, NFM was supplemented with 10 µm sodium molybdate (Na_2_MoO_4_) as a cofactor for nitrogenase. All cultures were incubated anaerobically at 30 °C under illumination from LED light (15 W, with a wavelength of 590–592 nm and light intensities of 60 µmol photons m^−2^s^−1^). During mutant construction, *R. palustris* strains were grown aerobically on PM agar plates supplemented with 10 mm succinate as the carbon and energy source at 30 °C in the dark. *Escherichia coli* strain S17‐1 was grown at 37 °C in Luria‐Bertani medium (pH 7). Where indicated, *R. palustris* was grown with 100 µg mL^−1^ gentamicin (Gm) while *E. coli* was grown with 10 µg mL^−1^ Gm.^[^ ^36^ ^]^

### Photosynthetic Bioelectrochemical Nitrogen Fixation (photo‐e‐BNF) System Construction and Operation

A two‐chamber H‐shaped cell was used to construct a three‐electrode system as previously described.^[^ ^72^ ^]^ Each chamber was designed with a liquid volume of 30 mL and a headspace volume of 25 mL. A proton exchange membrane (Nafion 117, DuPont Co., USA) was used to separate the two chambers. Carbon felts with dimensions of 2.5 cm × 2.5 cm were utilized as both anode and cathode. These were sequentially soaked in acetone, anhydrous ethanol, and deionized water, for 30 min each, and ultrasonically cleaned before use. A saturated calomel electrode (SCE), placed in the anode chamber, was employed as the reference electrode. The reactor was autoclaved at 121 °C for 20 min after assembly, and then the two chambers were filled with anaerobic PM or NFM medium to serve as electrolytes,^[^ ^73^ ^]^ except that the anolyte was supplemented with 20 mm sodium acetate. Cells at the logarithmic phase were collected and washed with PBS at least three times. Subsequently, the washed cells were inoculated into the anode chamber to achieve a final optical density at 660 nm (OD_660_) of 0.03. During operation, the working electrode was poised at +0.3 V (vs. SCE), and current data were simultaneously recorded by a multichannel electrochemical workstation (CHI 1040C, CH Instrument Inc., China). All experiments were carried out at least in triplicate at 30 °C under illuminated conditions unless otherwise indicated.

### Mutant Construction

In‐frame deletion mutants of *R. palustris* TIE‐1 were generated using the primers and plasmids listed in Tables S1 and S2 (Supporting Information). As previously described,^[^ ^32^, ^34^, ^35^ ^]^ regions ≈500 bp upstream and downstream of the gene to be deleted were amplified by PCR using high‐fidelity DNA polymerase (2 × Phanta Max Master Mix, Vazyme, China), with the chromosomal DNA of TIE‐1 (wild type) as a template. These two fragments were ligated to the linear plasmid of pJQ‐200SK using the In‐Fusion PCR cloning system, then maintained in *E. coli* S17‐1.^[^ ^74^ ^]^ For the creation of mutants XW001, XW009, and XW010, the constructed plasmids pJQ‐nifA^*^, pJQ‐*hupV*, and pJQ‐Δ*pioABC* were mobilized into TIE‐1 by conjugation with *E. coli* S17‐1. Colonies containing plasmids were identified by growth on PM agar plates containing Gm. Subsequently, these colonies were streaked onto PM plates supplemented with 10% sucrose and PM plates supplemented with 10% sucrose plus Gm, respectively. The recombinant strains were selected by sensitivity to gentamicin. For the in‐frame deletion of *cbbLSMP*, plasmid pJQ‐Δ*cbbLS* was transferred to TIE‐1 and XW001 by conjugation with *E. coli* S17‐1. The double crossover events for allelic exchange were achieved using the same protocol as described above. The obtained *R. palustris* XW003 and XW006 were used as the parent strains, and pJQ‐Δ*cbbM* was used as the suicide plasmid for the in‐frame deletion of *cbbM*, following the same process. Then, plasmid pJQ‐Δ*cbbP* was moved into *R. palustris* strains XW004 and XW007 for the next round of in‐frame deletion of *cbbP*, as described before. All mutations were verified by PCR and sequencing.

### Electrochemical Measurements

Differential pulse voltammetry (DPV) was conducted in a single‐chamber microbial electrolysis cell with an electrolyte solution of PBS (pH 7). A platinum sheet (1 cm × 2 cm) and SCE were used as the counter and reference electrodes, respectively. Biofilm DPV was conducted by placing the bioanode into the electrolyte. 0.22 µm filtered supernatant voltammograms were obtained using a glassy carbon electrode (3 mm in diameter). All electrochemical tests were conducted under a nitrogen atmosphere, and data were recorded using a CHI 1040C electrochemical workstation. The specific parameters were as follows: E_i_ = −0.8 V, E_f_ = 0.2 V, amplitude, 60 mV; pulse width, 250 ms; and potential increment, 6 mV. In some cases, dialysis bags (molecular mass cutoff of 1 kDa, Solarbio, China) were used to physically isolate cells and electrodes.^[^ ^75^ ^]^

### Nitrogen Fixation Characterization

^15^N‐labeling experiments were conducted to reflect the process of BNF. In detail, the headspace of the photo‐e‐BNF systems was filled with ^15^N‐labeled N_2_ (Acmec, China) after purging with high‐purity Ar (99.99%). Biofilms on the anode were collected by a vacuum drying process after their maturation. The ^15^N assimilation quantity was detected by an isotope mass spectrometer (MAT253, Thermo Fisher Scientific, USA).

Nitrogenase activity was measured via the acetylene (C_2_H_2_) reduction assay as previously described.^[^ ^16^ ^]^ The photo‐e‐BNF systems were purged with high‐purity Ar for at least 30 min after the current reached its peak. C_2_H_2_ was injected into the anodic headspace to reach a final concentration of 10% (v/v, gas phase). The concentration of ethylene (C_2_H_4_) was determined using a gas chromatograph (Shimadzu GC‐2014, Japan).

### Measurement of Intracellular ATP and NAD(P)H/NAD(P)^+^ Ratio

Cells from polarized and unpolarized anodes were collected when the current reached its maximum and then washed three times with resuspension and centrifugation (3 000 g, 5 min, 4 °C) in cold PBS solution (pH 7). For ATP quantitation, the Enhanced ATP bioluminescent assay kit (S0027, Beyotime Biotechnology, China) was used to measure ATP concentrations by detecting chemiluminescence with a luminometer plate reader. The final ATP concentrations were normalized to protein content. The NADPH/NADP^+^ ratio and NADH/NAD^+^ ratio were measured using the NADP/NADPH Quantification Kit (MAK038, Sigma‐Aldrich, USA) and NAD/NADH Quantification Kit (MAK037, Sigma‐Aldrich, USA), respectively, with a colorimetric assay read on a microplate reader at a wavelength of 450 nm.

### Analytical Techniques

Acetate and butyrate consumption were monitored by high‐performance liquid chromatography (HPLC, Shimadzu LC‐20A, Japan) with a UV detector set at 210 nm as described previously.^[^ ^76^ ^]^ When cultures of *R. palustris* strains in the photo‐e‐BNF systems reached their maximal OD_660_, H_2_ and CO_2_ (the sum of headspace CO_2_ plus dissolved CO_2_) were quantified with a Shimadzu GC‐2014 gas chromatograph as described previously.^[^ ^34^ ^]^

### Scanning Electron Microscopy (SEM)

Anodes were removed from the photo‐e‐BNF systems when the current reached its maximum level and immediately submerged in 2.5% glutaraldehyde. Samples were stored at 4 °C for 24 h before being dehydrated in 30%, 50%, 70%, 85%, 95%, and 100% ethanol in PBS solutions for 10 min each. Then, the samples were subjected to critical point drying with a 2 h purge time. After sputtering with a thin gold coating, the samples were pasted on the copper plate with carbon film tape and imaged using an SEM at 10 kV (Quanta FEG 450, USA).

### CLSM

The morphology of biofilms formed on the surface of anodes was examined with CLSM after the photo‐e‐BNF systems produced the maximum current density. The anode was rinsed briefly with sterile PBS to remove unattached cells and then stained with LIVE/DEAD™ *Bac*Light™ Bacterial Viability Kit (L7012, Thermo Fisher Scientific, USA) following the manufacturer's protocol. After incubation for 15 min in the dark, the sample was visualized under CLSM (Carl Zeiss LSM 980, Germany) at excitation wavelengths of 488 nm (SYTO 9) and 543 nm (propidium iodide).

### Transmission Electron Microscopy (TEM)

Biofilms from the anodes were collected at the end of the electricity generation experiment by centrifugation at 5 000 g for 5 min and immediately fixed in 2.5% glutaraldehyde in 50 mm HEPES at pH 7.4 on ice. After fixation, the samples were washed five times with resuspension and centrifugation (4 000 g, 2 min) in 50 mm HEPES buffer solution (pH 7.4) containing 35 g L^−1^ NaCl. Then, 0.5 mL of melted agarose was mixed with the sample by spinning at 5 000 rpm for 2 min. The mixed samples were kept on ice for 10 min and then sliced into small pieces (≈1 mm^3^). The detection of heme was performed with 3,3’‐diaminobenzidine (DAB) treatment, using previously described protocols.^[^ ^45^ ^]^ The reaction buffer solution containing 0.02% H_2_O_2_ and 0.0015 g mL^−1^ DAB was added to embedded sections for staining for 2.5 h at room temperature, while a DAB solution without H_2_O_2_ was added to parallel samples as a negative control. After staining, the DAB solution was removed by washing five times with 100 mm HEPES (pH 7.8). A 1% OsO_4_ solution (in 100 mm HEPES buffer) was added to each sample and incubated for 60 min on ice, followed by five washes with 1 mL of 100 mm HEPES (pH 8). The washed samples were dehydrated with a graded ethanol series (10 min each of 30%, 50%, 70%, 85%, 95%, 100%, 100%). The dehydrated samples were then treated with resin infiltration with a graded resin series (resin in acetone at 33.3%, 50%, 66.6%, 100% × 3 times) for 60 min each. Finally, the samples were transferred to a silicon mold filled with 100% resin and incubated at 65 °C for 24 h for polymerization. Thin sections of embedded samples were placed on copper microgrids, then examined and imaged using a FEI Tecnai Spirit TEM (Tecnai G2 F20 S‐TWIN, FEI, USA).

### Electron Shuttle Detection

Liquid chromatography (Thermo Ultimate 3000, USA) mass spectrometry (AB SCIEX™, TripleTOF5600+, USA) (LC‐MS) was performed to detect riboflavin and lumichrome in photo‐e‐BNF systems. Electrolyte samples were collected during the period of rapid current growth, then passed through a 0.22 µm pore‐size filter to remove cells. 1 mL of filtered electrolyte was dried with nitrogen and redissolved in 200 µL of methanol. The electrolyte extracts were injected at a flow rate of 0.15 mL min^−1^ for LC‐MS analysis by a reversed‐phase C18 column (GIST C18‐Aq, Shimadzu, Japan) maintained at 40 °C. A mobile phase comprised of 650 mm glacial acetic acid (phase A) and methanol (phase B) was used to elute riboflavin and lumichrome as previously reported.^[^ ^77^ ^]^

### Quantification of Biofilm and Planktonic Cells

The biomass in BESs was assessed by measuring protein content. The microbial cells on the anode were eluted using PBS solution (pH 7) and collected by centrifugation at 12 000 g for 10 min. Similarly, the anolyte containing planktonic cells was also subjected to centrifugation to collect the cells. After centrifugation, the cell pellets were stored at −80 °C overnight. Then, 1 mL of cell lysis buffer solution (containing 30 mm Tris‐HCl, pH 8.3, 500 mm NaCl, 5% Glycerol, and 1 mm PMSF) was added to the samples. Cells were lysed with a crusher (TAITEC, Japan) at 2 500 g for 30 s, followed by incubation on ice for 3 min. This process was repeated three times to ensure thorough lysis of the cells. The lysis supernatant was analyzed using the BCA Protein Assay Kit (C503021, Sangon Biotech, China). The total biomass was calculated as the sum of biofilm and plankton proteins.

### Transcriptomic Analysis

Three independent biological replicates with polarized/unpolarized were subjected to RNA‐seq analysis. Briefly, cells on the anode were collected when the current reached its peak. Total RNA was extracted using Invitrogen™ TRIzol™ Reagent (Thermo Fisher Scientific, USA). RNA integrity and concentration were measured using 1% agarose gel electrophoresis and NanoDrop 2000 (Thermo Fisher Scientific, USA). Transcriptome sequencing was performed at Majorbio Bio‐pharm Technology Co., Ltd. (Shanghai, China) using the Illumina HiSeq platform. Raw data were quality‐checked, filtered, and then mapped against the published reference genome of *Rhodopseudomonas palustris* TIE‐1 (CP001096.1). Gene transcription and expression levels were calculated using Salmon.^[^ ^78^ ^]^ Mapped reads were normalized with transcripts per million reads (TPM). Differences in gene expression between samples were analyzed using DESeq2 software based on the negative binomial distribution of raw counts,^[^ ^79^ ^]^ with the default parameters: *p*‐adjust < 0.05 and |log_2_FC| ≥ 1. All the raw sequencing data were deposited in NCBI Sequencing Read Archive under the accession number PRJNA1145169.

### Statistical Analysis

Statistical analyses were conducted using OriginPro 2023 (OriginLab, USA). Data were presented as mean ± standard deviation (SD) from three independent biological replicates (*n* = 3). Group comparisons were performed using a two‐tailed Student's *t*‐test at a significance level of *p* < 0.05. For all the analyses, *p < 0.05; **p < 0.01; ***p < 0.001; ns, not significant.

## Conflict of Interest

The authors declare no competing interests.

## Supporting information



Supporting Information

Supporting Information

Supporting Information

## Data Availability

The data that support the findings of this study are available from the corresponding author upon reasonable request.
