# Supplementary material for: Non‐Respiratory Extracellular Electron Transfer Competes with Nitrogenase for Electrons in Rhodopseudomonas Palustris
Source: Adv Sci (Weinh). 2025 May 14;12(25):2501376. doi: 10.1002/advs.202501376 (PMC12225005; doi:10.1002/advs.202501376)
Supplement: Supplementary file 1 — Supporting Information [file ADVS-12-2501376-s001.docx]

Supporting Information

**Non-Respiratory Extracellular Electron Transfer Competes with Nitrogenase for Electrons in *Rhodopseudomonas palustris***

Xuewen Liu, Panqing Qi, Weipeng Fan, Wuyang Liu, Xingjiang Li, Yong Nie*, Xiao-Lei Wu*

**Table S1** **Strains and plasmids used in this study.**

| Strain or plasmid | Genotype or phenotypea^a)^ | Source or reference |
| --- | --- | --- |
| *R. palustris* TIE-1 | Wild-type (WT) | Institute of Microbiology, Chinese Academy of Sciences, Yanning zheng |
| *R. palustris* XW001 | 48 bp deletion in NifA Q-linker region, nifA^*^ | This work |
| *R. palustris* XW003 | WTΔ*cbbLS* | This work |
| *R. palustris* XW004 | WTΔ*cbbLSM* | This work |
| *R. palustris* XW005 | WTΔ*cbbLSMP* | This work |
| *R. palustris* XW006 | nifA^*^Δ*cbbLS* | This work |
| *R. palustris* XW007 | nifA^*^Δ*cbbLSM* | This work |
| *R. palustris* XW008 | nifA^*^Δ*cbbLSMP* | This work |
| *R. palustris* XW009 | 4 bp deletion in *hupV*, WT*hupV*^-^ | This work |
| *R. palustris* XW010 | WTΔ*pioABC* | This work |
| *E. coli* S17-1 | *pro hdsR recA*; chromosomal insertion of RP4-2 (Tc::Mu Km^r^::Tn7) | ^[1]^ |
| pJQ200SK | Gm^r^, sacB, mobilizable suicide vector | ^[2]^ |
| pJQ-nifA^*^ | Gm^r^, in-frame nifA^*^cloned into pJQ200SK | This work |
| pJQ-Δ*cbbLS* | Gm^r^, in-frame Δ*cbbLS* cloned into pJQ200SK | This work |
| pJQ-Δ*cbbM* | Gm^r^, in-frame Δ*cbbM* cloned into pJQ200SK | This work |
| pJQ-Δ*cbbP* | Gm^r^, in-frame Δ*cbbP* cloned into pJQ200SK | This work |
| pJQ-*hupV* | Gm^r^, in-frame *hupV* cloned into pJQ200SK | This work |
| pJQ-Δ*pioABC* | Gm^r^, in-frame Δ*pioABC* cloned into pJQ200SK | This work |

^a)^ Km^r^, kanamycin resistance; Gm^r^, gentamicin resistance.

**Table S2 Primers used in this study**

| Primers | Sequence (5’–3’) | Source |
| --- | --- | --- |
| NifA^*^-upF | GTCGTGCCAGGGGAATTAATGCAGGATCATCGGCACTAC | This work |
| NifA^*^-upR | CGACCGCGAGCGGCTGGAGCGCGAGCGCAAGC | This work |
| NifA^*^-dnF | GCTTGCGCTCGCGCTCCAGCCGCTCGCGGTCG | This work |
| NifA^*^-dnR | AGTAGCTGAACAGGAGGGACAGACTCACCGGCATTTTCGAGA | This work |
| *cbbLS*-upF | GTCGTGCCAGGGGAATTAATGATGTTTGCCAGCGAGCAC | This work |
| *cbbLS*-upR | GCTGTCGCGTCCACGAGTCGTCCTCCTTGAAAGCC | This work |
| *cbbLS*-dnF | GGCTTTCAAGGAGGACGACTCGTGGACGCGACAGC | This work |
| *cbbLS*-dnR | AGTAGCTGAACAGGAGGGACAGACCATCACCTGGAGCAGGATC | This work |
| *cbbM*-upF | GTCGTGCCAGGGGAATTAATGATGAGCGAGCCGCTCC | This work |
| *cbbM*-upR | CGCATTGCAGGAGATCACCGCTGGCCTAGTCGACACG | This work |
| *cbbM*-dnF | CGTGTCGACTAGGCCAGCGGTGATCTCCTGCAATGCG | This work |
| *cbbM*-dnR | AGTAGCTGAACAGGAGGGACAGAGACGTGCTGGCCATGAAG | This work |
| *cbbP*-upF | GTCGTGCCAGGGGAATTAATCGATGACGTAGGTGTGGTGAT | This work |
| *cbbP*-upR | GTTGGAATTGGAAGTAACTCCTCTGCCCAACATCTGCAAGAAT | This work |
| *cbbP*-dnF | ATTCTTGCAGATGTTGGGCAGAGGAGTTACTTCCAATTCCAAC | This work |
| *cbbP*-dnR | AGTAGCTGAACAGGAGGGACAGACGACACCGCCGAATACG | This work |
| *hupV*-upF | GTCGTGCCAGGGGAATTAATTTCTTCATGCCGGACTTCG | This work |
| *hupV*-upR | CTCGGTGATCGCGGCAGGAACGACGTCCGCGCCGCACTTAAT | This work |
| *hupV*-dnF | ATTAAGTGCGGCGCGGACGTCGTTCCTGCCGCGATCACCGAG | This work |
| *hupV*-dnR | AGTAGCTGAACAGGAGGGACAGAGACCAGCGCTTGTTCGAGC | This work |
| *pio*-upF | GTCGTGCCAGGGGAATTAATACCGCACCAAGGACATGTAC | This work |
| *pio*-upR | ACTTGCGGGGGACGGACGCGCCGTTGATCACG | This work |
| *pio*-dnF | CGTGATCAACGGCGCGTCCGTCCCCCGCAAGT | This work |
| *pio*-dnR | AGTAGCTGAACAGGAGGGACAGAGACAATCAGTCGACCCGACC | This work |

**
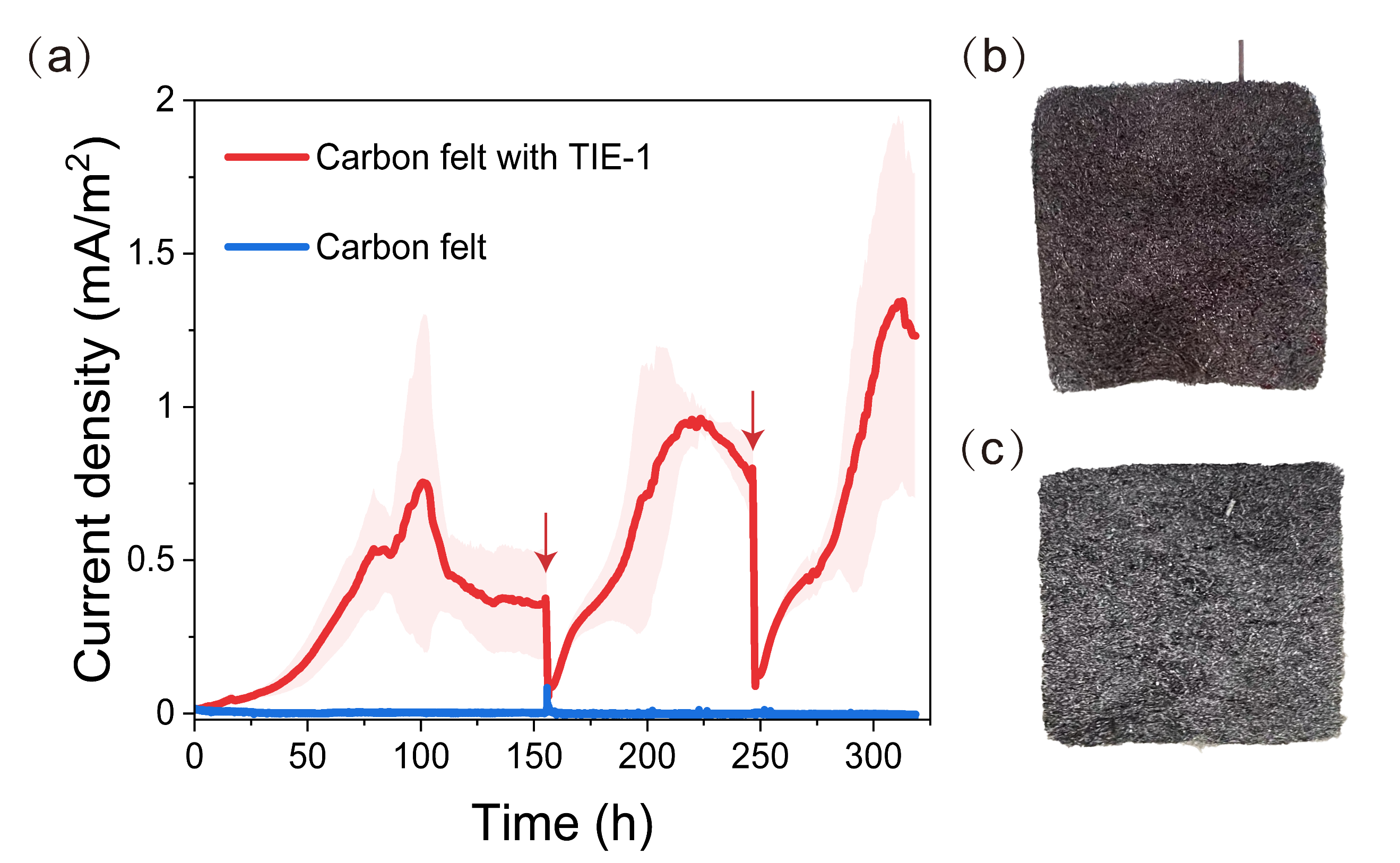
**

**Figure S1.** (a) Current generation in continuous mode. The red arrows indicate the replacement of fresh electrolyte. The shaded area represents the standard deviation (n = 3 biological replicates). Anodes from (b) biotic and (c) abiotic groups.


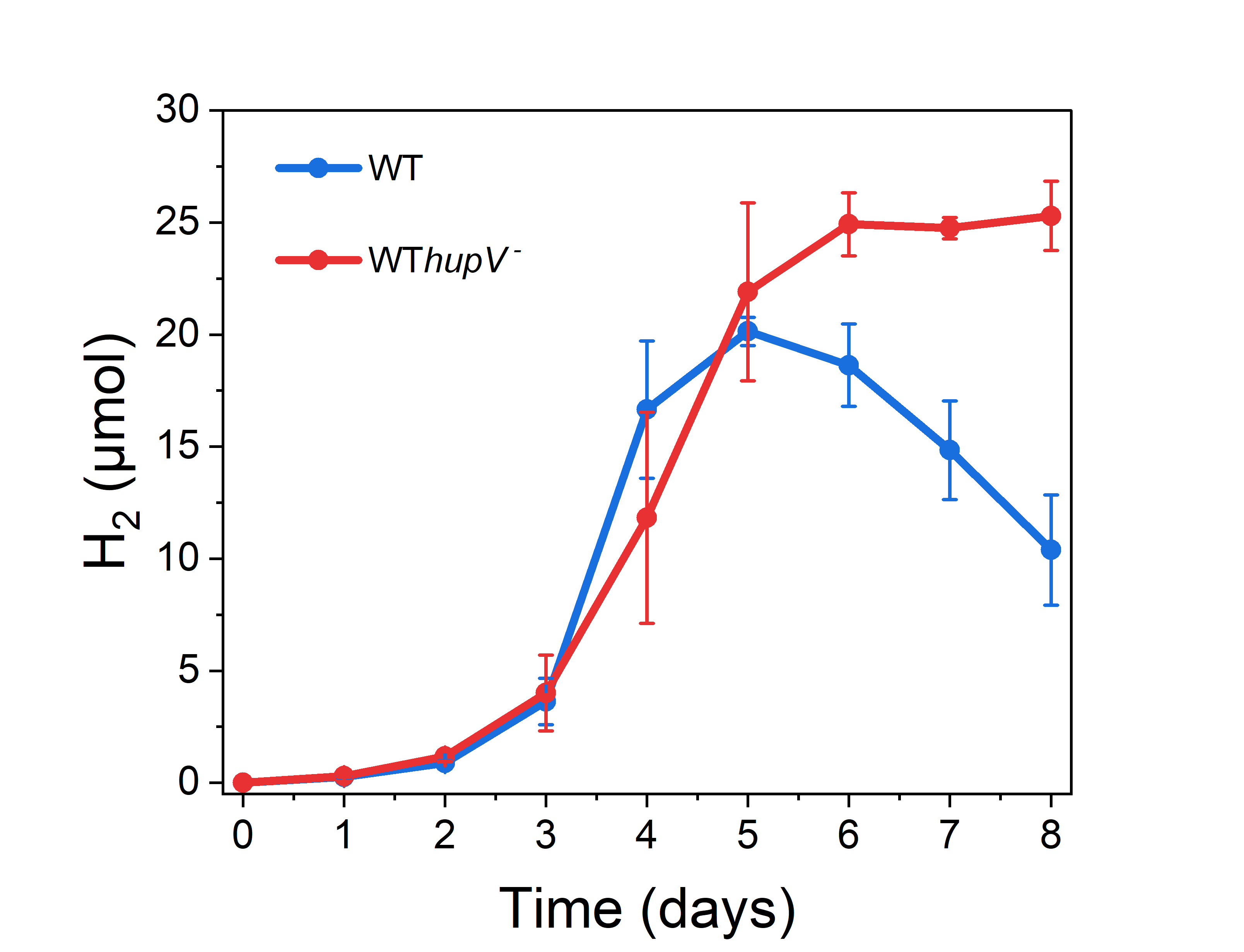


**Figure S2**. The hydrogen accumulation of WT and WT*hupV*^-^. Cultures were grown anaerobically in a sealed 50 mL anaerobic culture bottle with 20 mL NFM. Data were presented as mean ± SD (n = 3).


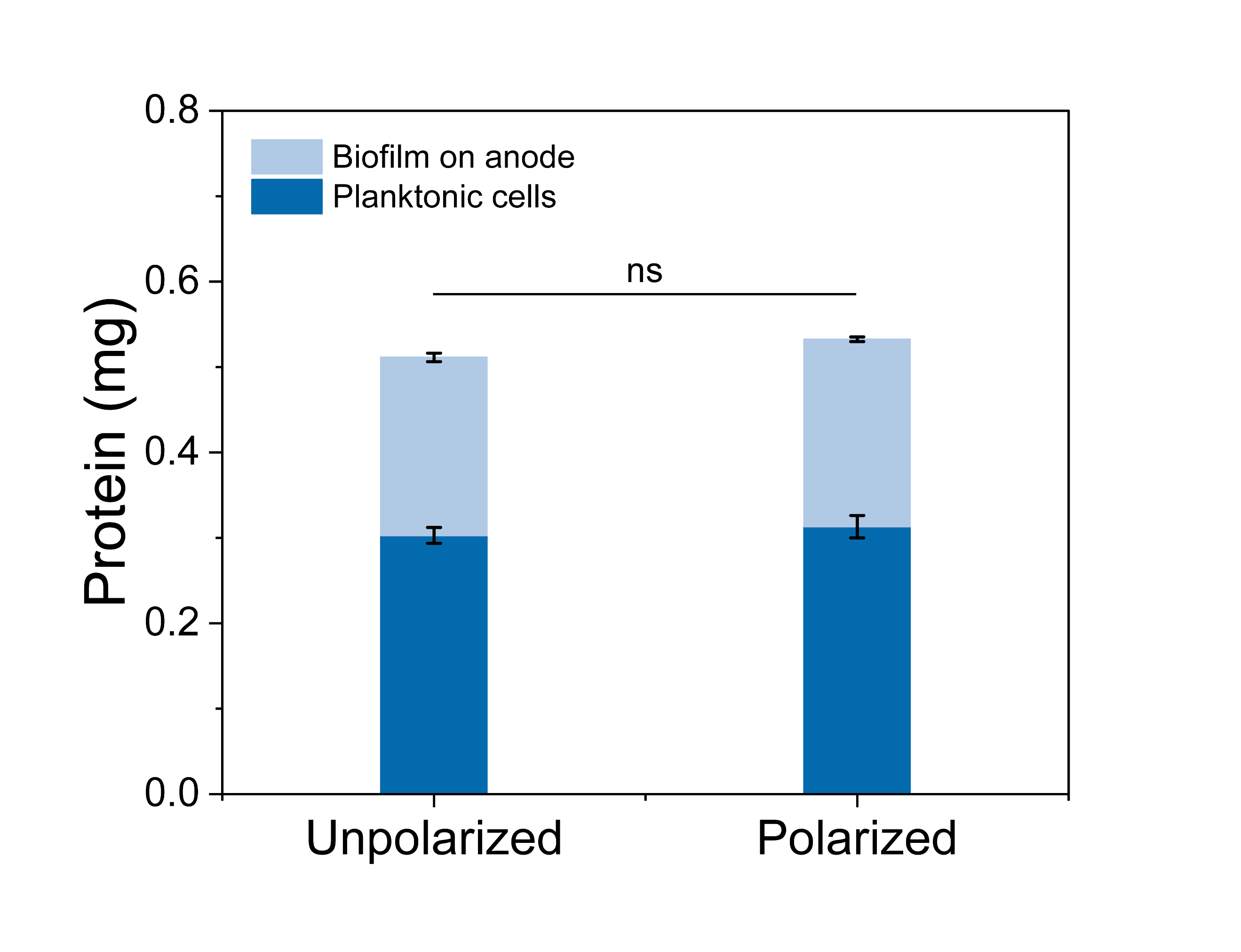


**Figure S3.** Biomass of WT*hupV*^-^ in photo-e-BNF systems under unpolarized and polarized conditions. Data were presented as mean ± SD (n = 3). Statistical analysis was performed using two-tailed Student’s *t*-tests. ns, no significance.


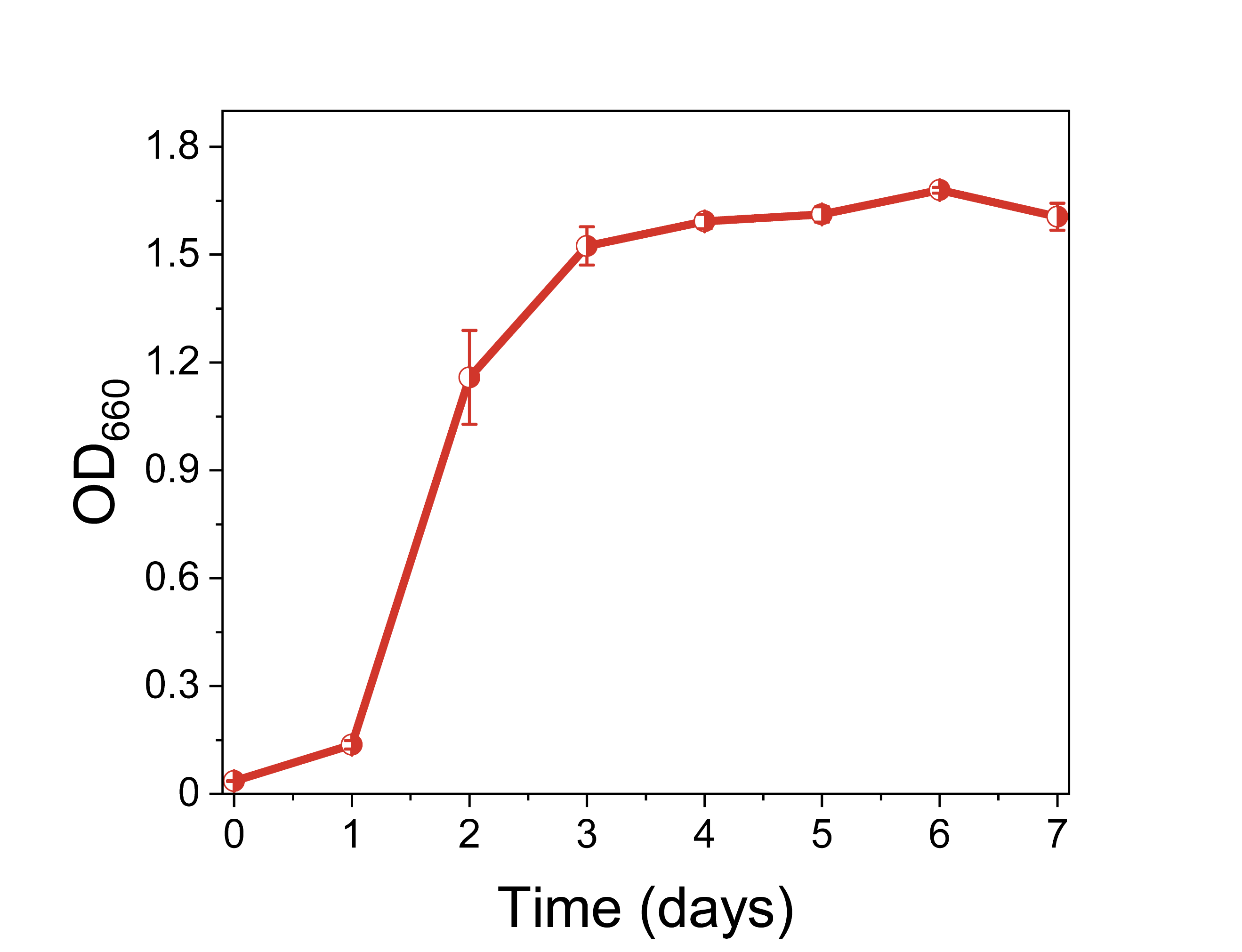


**Figure S4.** The growth curve of *R. palustris* TIE-1 under nitrogen-fixation conditions in the absence of poised anodes. Data were presented as mean ± SD (n = 3).


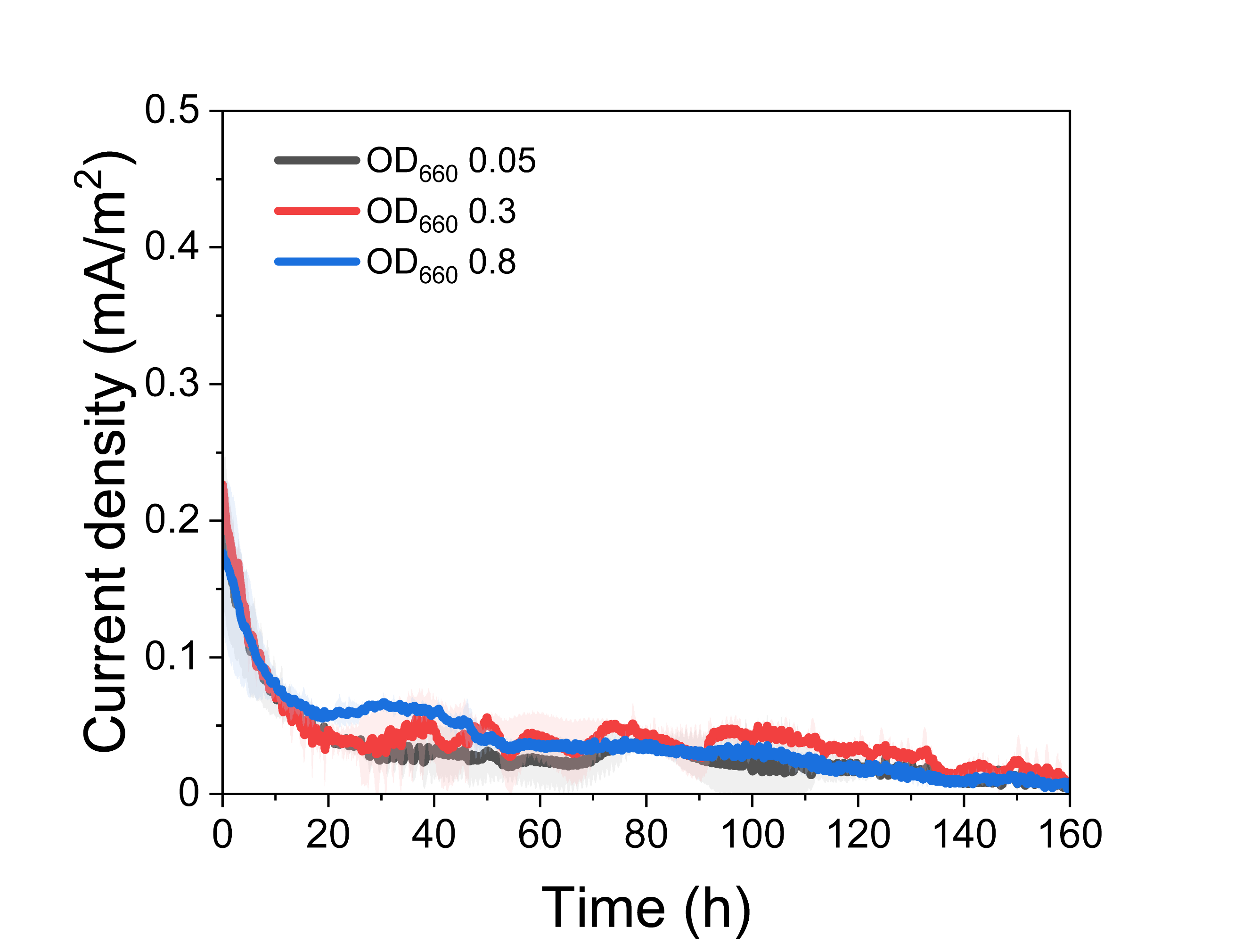


**Figure S5.** The current curves of *R. palustris* TIE-1 at different inoculation concentrations in the absence of electron donors under illumination conditions. The shaded area represents the standard deviation (n = 3 biological replicates).


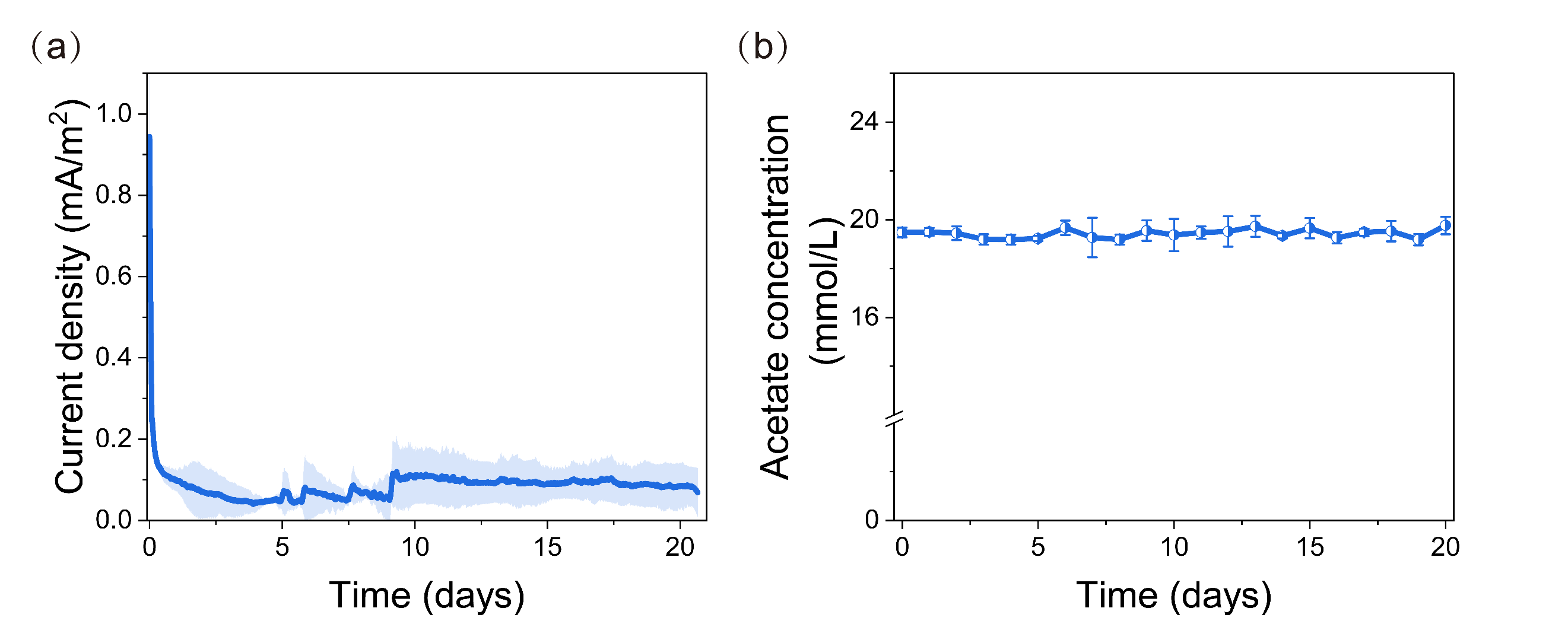


**Figure S6.** (a) Current curves and (b) acetate consumption profiles of e-BNF systems under dark conditions over an extended operational period. Data were presented as mean ± SD (n = 3).


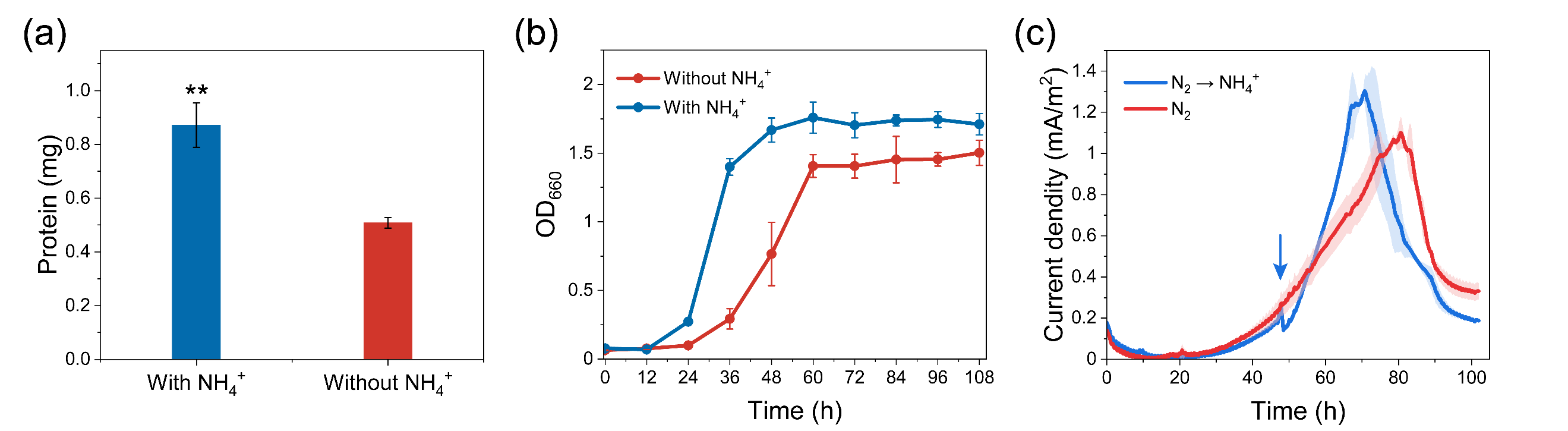


**Figure S7.** (a) Total biomass of BESs under N-sufficient and N-deficient conditions. (b) Growth curves of *R. palustris* TIE-1 under N-sufficient and N-deficient conditions. (c) Current generation by *R. palustris* TIE-1 under nitrogen fixation conditions and with the addition of ammonium during nitrogen fixation. The blue arrow indicates the addition of ammonium. Data were presented as mean ± SD (n = 3). Statistical analysis was performed using two-tailed Student’s *t*-tests (a). ** *p*<0.01.


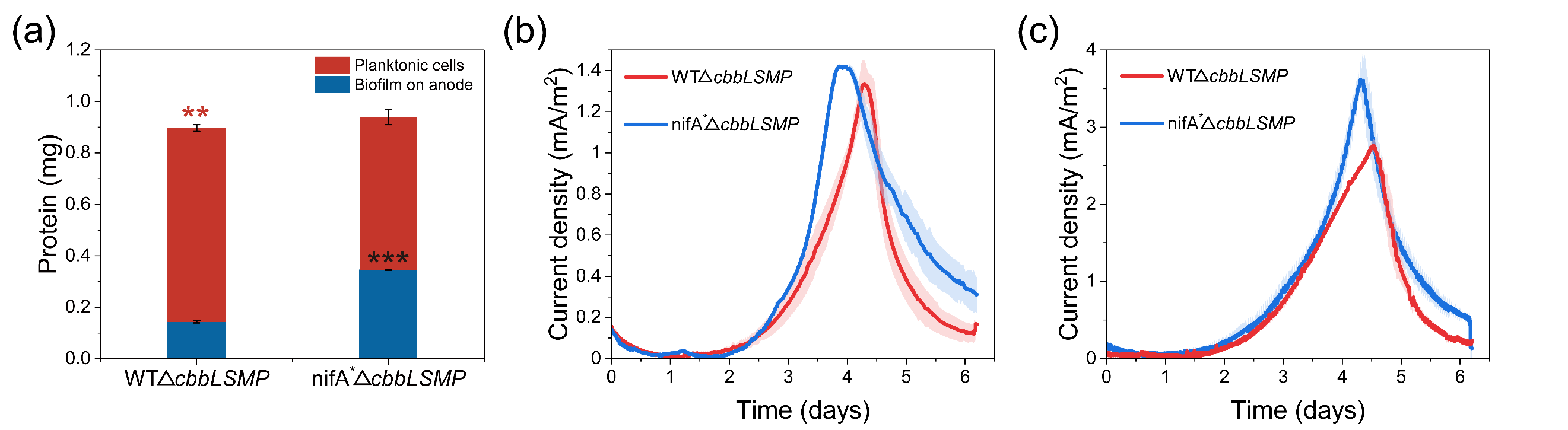


**Figure S8.** (a) Biomass of WTΔ*cbbLSMP* and nifA^*^Δ*cbbLSMP* mutants in photo-e-BNF systems. (b) Current generation with anodes shielded from WTΔ*cbbLSMP* and nifA^*^Δ*cbbLSMP* mutants by a dialysis membrane. (c) Current generation by WTΔ*cbbLSMP* and nifA^*^Δ*cbbLSMP* mutants. Data were presented as mean ± SD (n = 3). Statistical analysis was performed using two-tailed Student’s *t*-tests (a). ** *p*<0.01, *** *p*<0.001.


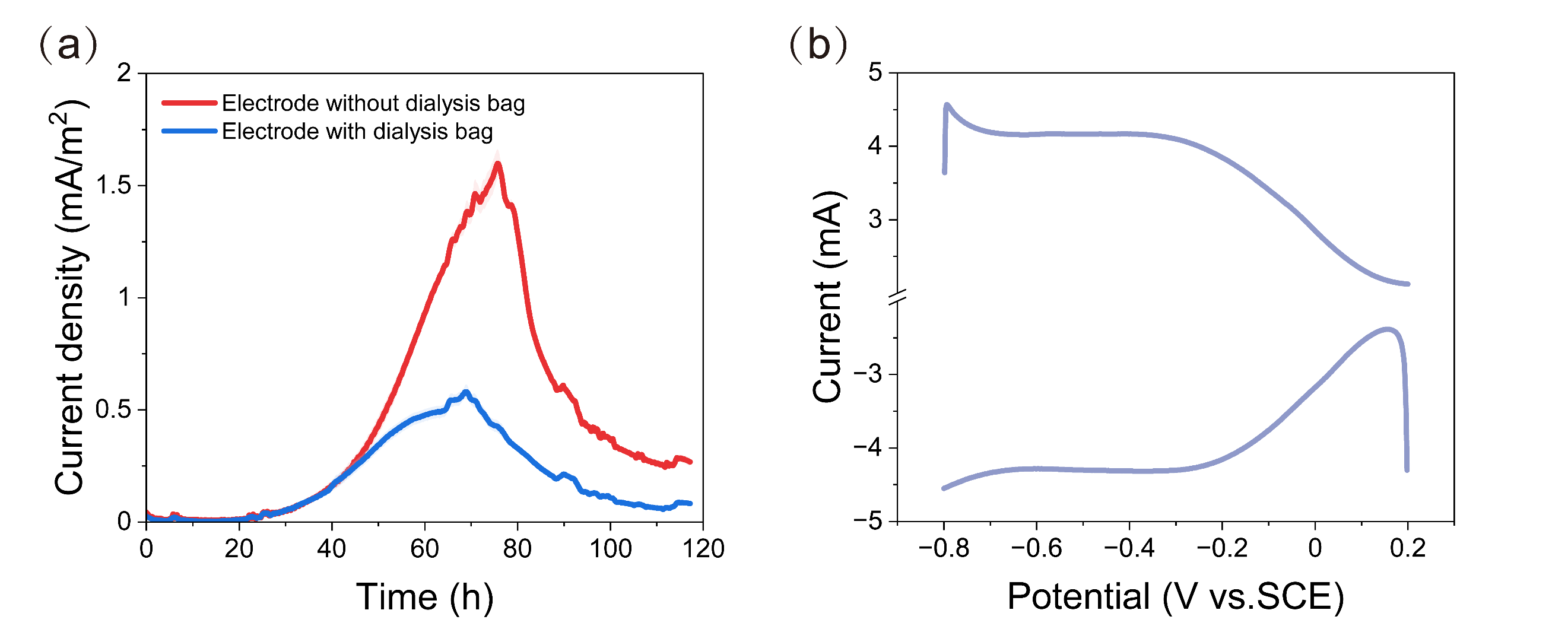


**Figure S9.** (a) Current curve of photo-e-BNF systems with electrodes isolated or non-isolated with *R. palustris* TIE-1 cells by dialysis bags. (b) DPV analyses of anodes from abiotic control. The shaded area represents the standard deviation (n = 3 biological replicates).


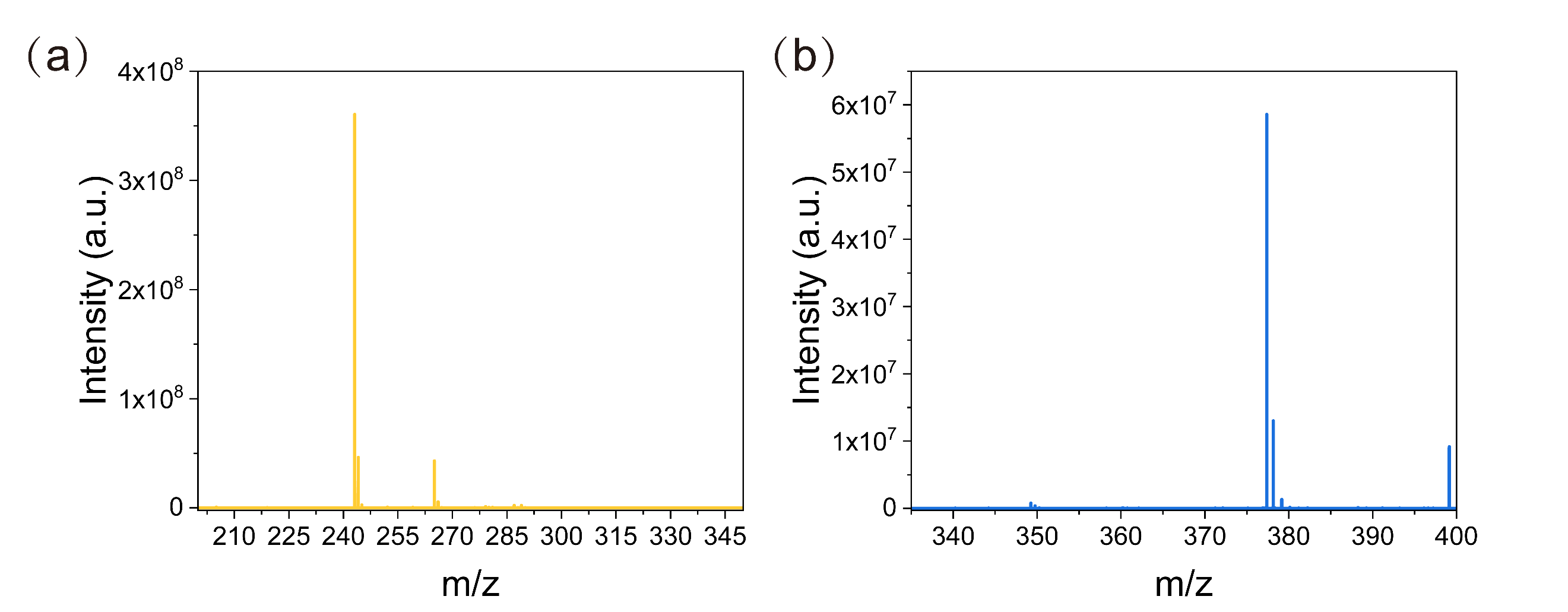


**Figure S10.** Mass spectrum of (a) lumichrome and (b) riboflavin standard substances.


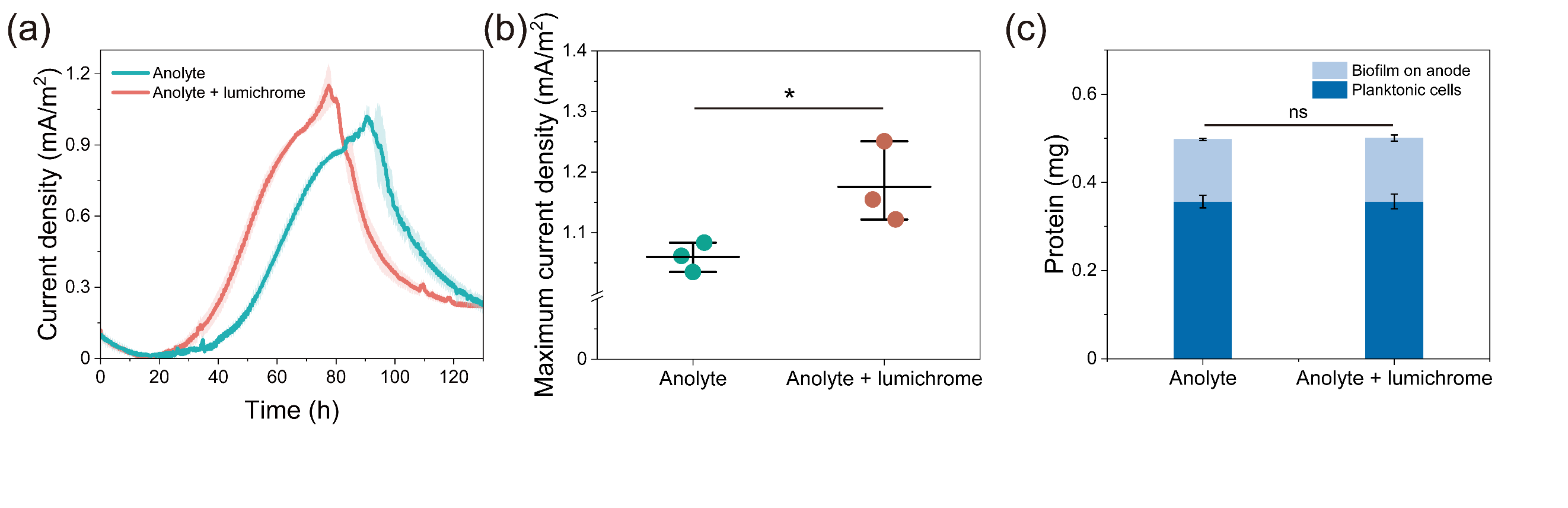


**Figure S11.** Current generation (a, b) and biomass (c) of photo-e-BNF systems with or without the addition of lumichrome as electron shuttle. Data were presented as mean ± SD (n = 3). Statistical analysis was performed using two-tailed Student’s *t*-tests (b, c). * *p*<0.05, ns, no significance.


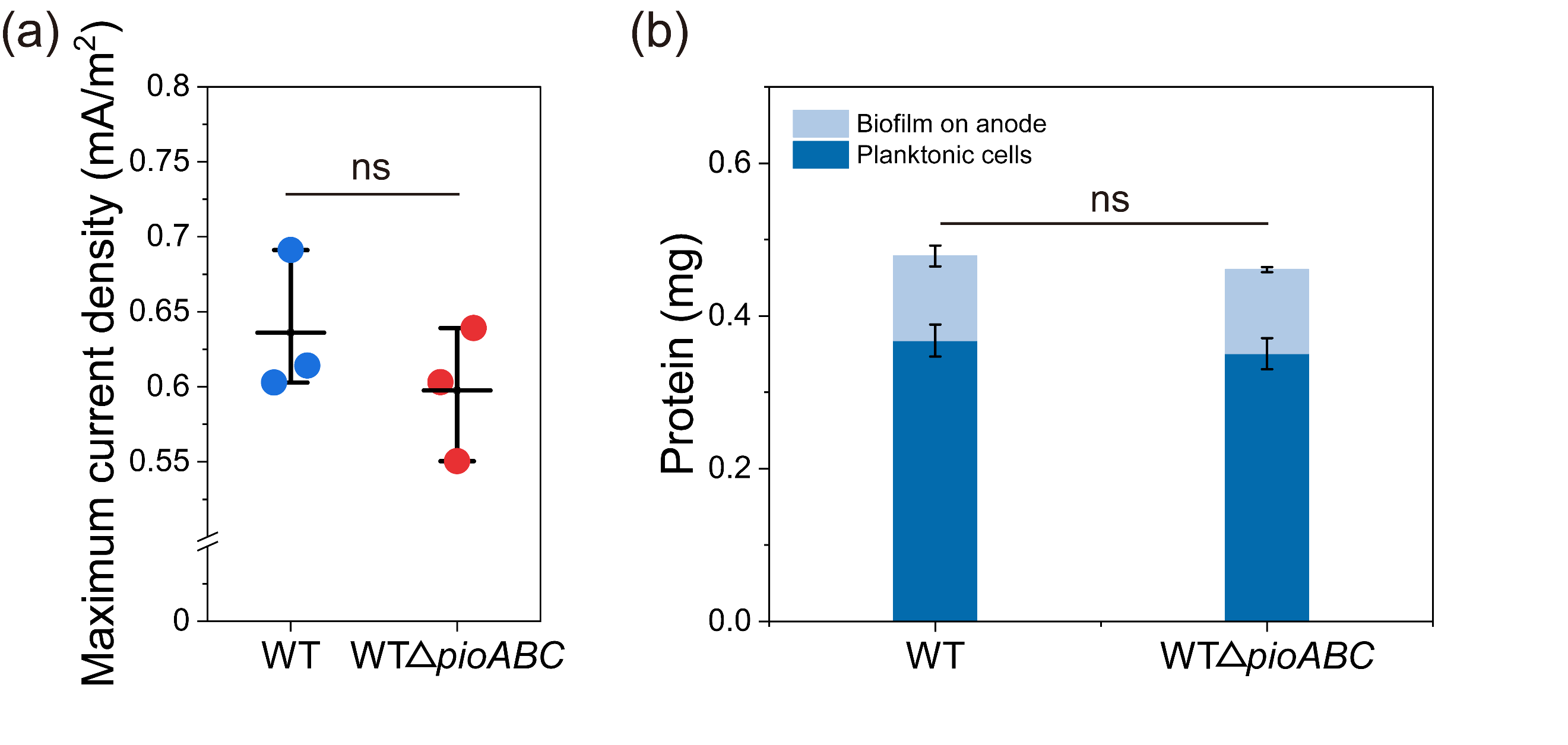


**Figure S12.** (a) Maximum current density of photo-e-BNF systems with electrodes isolated with WT and WTΔ*pioABC* by dialysis bags. (b) Biomass of WT and WTΔ*pioABC* in photo-e-BNF systems. Data were presented as mean ± SD (n = 3). Statistical analysis was performed using two-tailed Student’s *t*-tests. ns, no significance.


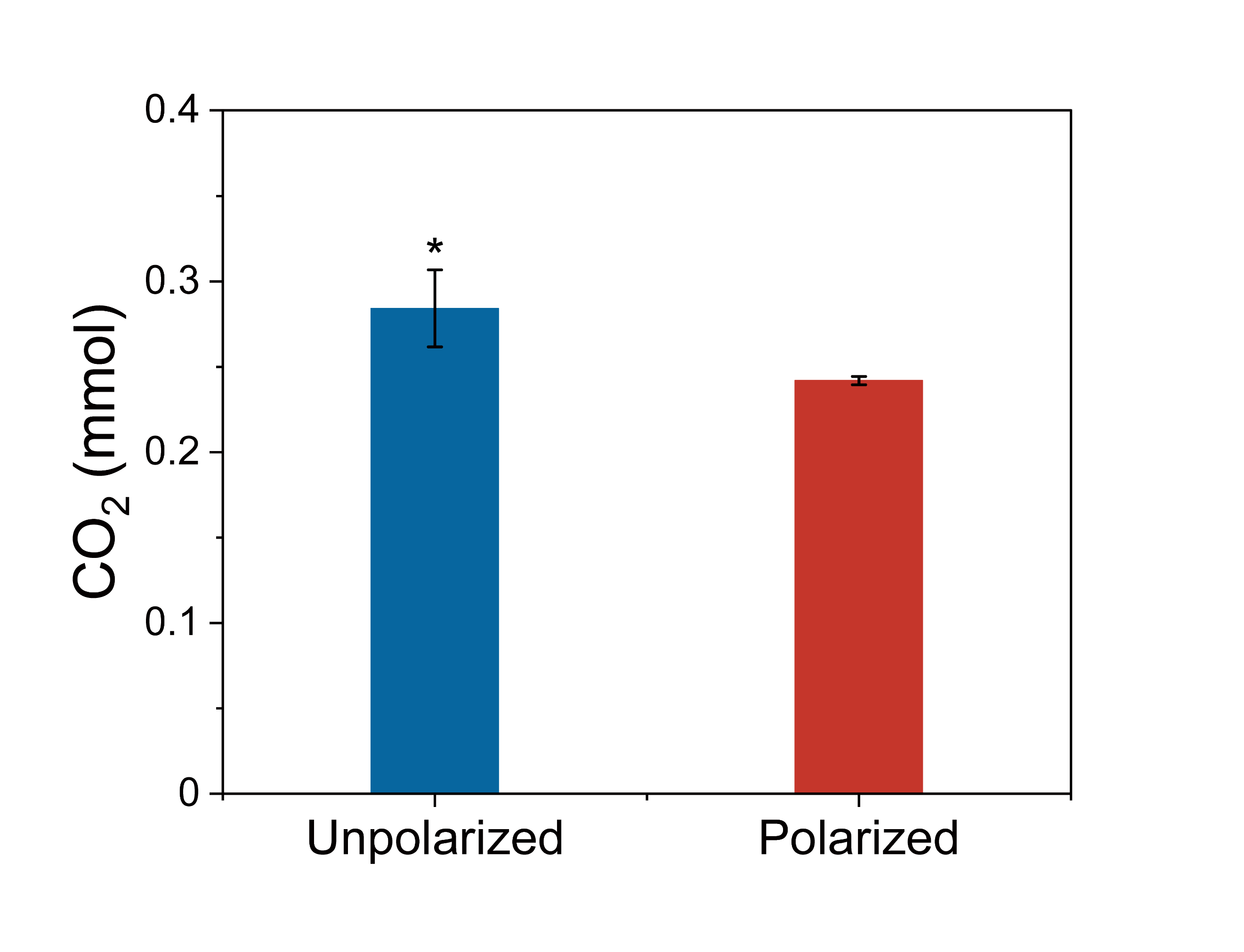


**Figure S13.** Accumulation of CO_2_ by WT*hupV*^-^ in photo-e-BNF systems with or without poised anodes. Data were presented as mean ± SD (n = 3). Statistical analysis was performed using two-tailed Student’s *t*-tests. * *p*<0.05.

**Reference**

[1] R. Simon, U. Priefer, A. Pühler, A broad host range mobilization system for in vivo genetic engineering: transposon mutagenesis in gram negative bacteria, *Bio/technology* **1983**, *1* (9), 784.

[2] J. Quandt, M. F. Hynes, Versatile suicide vectors which allow direct selection for gene replacement in gram-negative bacteria, *Gene* **1993**, *127* (1), 15.
